# Supplementary material for: Stigma experiences and adaptations in accessing healthcare services among hill tribes in Thailand: A qualitative study
Source: PLoS One. 2025 May 2;20(5):e0321119. doi: 10.1371/journal.pone.0321119 (PMC12047764; doi:10.1371/journal.pone.0321119)
Supplement: S1 File — (DOCX) [file pone.0321119.s001.docx]

**Question guide**

1) Have you ever experienced stigma while attending a hospital?

2) Could you please give me details of the stigma you experienced?

3) Who caused the stigma against you—a doctor, nurse or someone else?

4) How frequently did you experience stigma?

5) How did you feel while experiencing stigma?

6) How did you respond to the event?

7) Did it impact your health?

8) Why did you not receive good service from the health care provider?

9) What expectations did you have before accessing the health care service?
