# Supplementary material for: Stigma experiences and adaptations in accessing healthcare services among hill tribes in Thailand: A qualitative study
Source: PLoS One. 2025 May 2;20(5):e0321119. doi: 10.1371/journal.pone.0321119 (PMC12047764; doi:10.1371/journal.pone.0321119)
Supplement: Attachment — Submitted filename: PONE-D-23-10436.pdf [file pone.0321119.s002.pdf]

PLOS ONE

Stigma experiences and adaptations while accessing health care service among hill tribes in Thailand: a qualitative study

--Manuscript Draft--

**Manuscript Number:**	PONE-D-23-10436
**Article Type:**	Research Article
**Full Title:**	Stigma experiences and adaptations while accessing health care service among hill tribes in Thailand: a qualitative study
**Short Title:**	Stigma experiences and adaptations while accessing health care service among hill tribes in Thailand
**Corresponding Author:**	Tawatchai Apidechkul, Dr.P.H.(Epidemiology)<br>Mae Fat Luang University<br>Chiang Rai, THAILAND
**Keywords:**	stigma; Experiences; Hill tribe; Access to public health and medical services
**Abstract:**	Background<br><br>One of the major barriers to accessing health care services is experiencing stigma from healthcare workers. Individuals can be greatly impacted by stigma owing to be classed according to particular characteristics, such as being a tribal member. This study aimed to understand the experiences and adaptations of hill tribe people in Thailand who faced stigma when accessing health care services.<br><br>Methods<br><br>A phenomenological qualitative method was used to elicit information from hill tribe members who had prior experience in accessing health care. A question guide was used to interview the participants. Interviews were conducted in hill tribe villages in a private and confidential room in August 2021. Each interview lasted for 45 minutes.<br><br>Results<br><br>A total of 85 people participated in the study: 25 men and 60 women. Akha and Lahu people were the majority. A large proportion had no education and the average monthly income was 2,500 baht per family. Three forms of stigma were detected among the hill tribe people while accessing health care services in different hospitals: verbal stigma, physical stigma, and negative looks. Three levels of impacts were found: completely not understood with no impact, little understanding with little pain, and fully understood with full impact. Two response approaches were identified: nonresponse and response (proper, nonproper immediate response, and assertive response). Three factors were protective against stigma: speaking fluent Thai, wearing modern clothing, and the ability to pay medical fees.<br><br>Conclusions<br><br>Hill tribe people face several forms of stigma. This results in various levels of impact and different response methods. Some factors can be protective against stigma while accessing health care services in a hospital. Implementing a program to reduce stigma should be focused on these factors to improve the level of access to care among the hill tribe population.
**Order of Authors:**	Peeradone Srichan
	Tawatchai Apidechkul, Dr.P.H.(Epidemiology)
	Ratipark Tamornpark
	Thanatchaporn Mulikaburt
	Pilasinee Wongnuch

Powered by Editorial Manager® and ProduXion Manager® from Aries Systems Corporation

	Siwarak Kitchanapaibul
	Panupong Upala
	Chalitar Chomchoei
	Fartima Yeemard
	Anusorn Udplong
	Onnalin Singkhorn
**Additional Information:**	
**Question**	**Response**
**Financial Disclosure**<br><br>Enter a financial disclosure statement that describes the sources of funding for the work included in this submission. Review the submission guidelines for detailed requirements. View published research articles from *PLOS ONE* for specific examples.<br><br>This statement is required for submission and **will appear in the published article** if the submission is accepted. Please make sure it is accurate.<br><br>**Unfunded studies**<br>Enter: *The author(s) received no specific funding for this work.*<br><br>**Funded studies**<br>Enter a statement with the following details:<br>• Initials of the authors who received each award<br>• Grant numbers awarded to each author<br>• The full name of each funder<br>• URL of each funder website<br>• Did the sponsors or funders play any role in the study design, data collection and analysis, decision to publish, or preparation of the manuscript?<br>• **NO** - Include this sentence at the end of your statement: *The funders had no role in study design, data collection and analysis, decision to publish, or preparation of the manuscript.*<br>• **YES** - Specify the role(s) played.<br><br>* typeset	The funders had no role in study design, data collection and analysis, decision to publish, or preparation of the manuscript.
**Competing Interests**<br><br>Use the instructions below to enter a	The authors have declared that no competing interests exist.

competing interest statement for this submission. On behalf of all authors, disclose any competing interests that could be perceived to bias this work—acknowledging all financial support and any other relevant financial or non-financial competing interests.<br><br>This statement is required for submission and **will appear in the published article** if the submission is accepted. Please make sure it is accurate and that any funding sources listed in your Funding Information later in the submission form are also declared in your Financial Disclosure statement.<br><br>View published research articles from *PLOS ONE* for specific examples.<br><br>**NO authors have competing interests**<br><br>Enter: *The authors have declared that no competing interests exist.*<br><br>**Authors with competing interests**<br><br>Enter competing interest details beginning with this statement:<br><br>*I have read the journal's policy and the authors of this manuscript have the following competing interests: [insert competing interests here]*<br><br>* typeset	
**Ethics Statement**<br><br>Enter an ethics statement for this submission. This statement is required if the study involved:<br><br>• Human participants<br>• Human specimens or tissue<br>• Vertebrate animals or cephalopods<br>• Vertebrate embryos or tissues<br>• Field research	All research protocols, including the question guide, were approved by the Chiang Rai Provincial Public Health Office (CRPPHO No. 69/2564). All activities were performed in accordance with the relevant guidelines and regulations. Before the interviews, all participants were provided information about the study and asked for their informed voluntary consent. For those who did not understand Thai, a relative who was fluent in Thai was asked to interpret before fingerprinting the informed consent form. For those younger than 18 years, informed consent was also obtained from their parents before the interviews. All records were properly destroyed after completion of the analysis. Information from the study was presented anonymously without being able to identify an individual's details.

Powered by Editorial Manager® and ProduXion Manager® from Aries Systems Corporation

Write "N/A" if the submission does not require an ethics statement.

General guidance is provided below. Consult the submission guidelines for detailed instructions. **Make sure that all information entered here is included in the Methods section of the manuscript.**

Format for specific study types

Human Subject Research (involving human participants and/or tissue)

- Give the name of the institutional review board or ethics committee that approved the study
- Include the approval number and/or a statement indicating approval of this research
- Indicate the form of consent obtained (written/oral) or the reason that consent was not obtained (e.g. the data were analyzed anonymously)

Animal Research (involving vertebrate animals, embryos or tissues)

- Provide the name of the Institutional Animal Care and Use Committee (IACUC) or other relevant ethics board that reviewed the study protocol, and indicate whether they approved this research or granted a formal waiver of ethical approval
- Include an approval number if one was obtained
- If the study involved *non-human primates*, add *additional details* about animal welfare and steps taken to ameliorate suffering
- If anesthesia, euthanasia, or any kind of animal sacrifice is part of the study, include briefly which substances and/or methods were applied

Field Research

Include the following details if this study involves the collection of plant, animal, or other materials from a natural setting:

- Field permit number
- Name of the institution or relevant body that granted permission

Powered by Editorial Manager® and ProduXion Manager® from Aries Systems Corporation

**Data Availability**<br><br>Authors are required to make all data underlying the findings described fully available, without restriction, and from the time of publication. PLOS allows rare exceptions to address legal and ethical concerns. See the PLOS Data Policy and FAQ for detailed information.<br><br>A Data Availability Statement describing where the data can be found is required at submission. Your answers to this question constitute the Data Availability Statement and **will be published in the article**, if accepted.<br><br>**Important:** Stating 'data available on request from the author' is not sufficient. If your data are only available upon request, select 'No' for the first question and explain your exceptional situation in the text box.<br><br>Do the authors confirm that all data underlying the findings described in their manuscript are fully available without restriction?	Yes - all data are fully available without restriction
**Describe where the data may be found in full sentences. If you are copying our sample text, replace any instances of XXX with the appropriate details.**<br><br>• If the data are **held or will be held in a public repository**, include URLs, accession numbers or DOIs. If this information will only be available after acceptance, indicate this by ticking the box below. For example: *All XXX files are available from the XXX database (accession number(s) XXX, XXX.*).<br>• If the data are all contained **within the manuscript and/or Supporting Information files**, enter the following: *All relevant data are within the manuscript and its Supporting Information files.*<br>• If neither of these applies but you are able to provide **details of access elsewhere**, with or without limitations, please do so. For example:	All relevant data are within the manuscript and its Supporting Information files.

Data cannot be shared publicly because of [XXX]. Data are available from the XXX Institutional Data Access / Ethics Committee (contact via XXX) for researchers who meet the criteria for access to confidential data.<br><br>*The data underlying the results presented in the study are available from (include the name of the third party and contact information or URL).*<br>• This text is appropriate if the data are owned by a third party and authors do not have permission to share the data.<br><br>* typeset	
Additional data availability information:	

Powered by Editorial Manager® and ProduXion Manager® from Aries Systems Corporation

Manuscript

Click here to access/download;Manuscript;Stigma-Peeradone.docx

Stigma experiences and adaptations while accessing health care service among hill tribes in Thailand: a qualitative study

Peeradone Srichan[1,2,] Tawatchai Apidechkul[1,2*], Ratipark Tamornpark[1,2], Thanatchaporn Mulikaburt[1], Pilasinee Wongnuch[1,2], Siwarak Kitchanapaibul[1,2], Panupong Upala[2], Chalitar Chomchoei[2], Fartima Yeemard[2], Anusorn Udplong[1], Onnalin Singkhorn[2,3]

[1]School of Health Sciences, Mae Fah Luang University, Chiang Rai, Thailand

[2]Center of Excellence for Hill Tribe Health Research, Mae Fah Luang University, Chiang Rai, Thailand

[3]School of Nursing, Mae Fah Luang University, Chiang Rai, Thailand

Email authors:

Peeradone Srichan email: Peeradone.sri@mfu.ac.th

Tawatchai Apidechkul email: Tawatchai.api@mfu.ac.th

Ratipark Tamornpark email: Ratipark.tam@mfu.ac.th

Thanatchaporn Mulikaburt email: Thanatchaporn.mul@mfu.ac.th

Pilasinee Wongnuch email: Pilasinee.won@mfu.ac.th

Siwarak Kitchanapaibul email: Siwarak@mfu.ac.th

Panupong Upala email: panupong.upa@mfu.ac.th

Chalitar Chomchoei email: chalitar.cho13@lamduan.mfu.ac.th

Fartima Yeemard email: Fartima.far052@gmail.com

Anusorn Udplong email: Anusorn.udp@mfu.ac.th

Onnalin Singkhorn email: onnalin.sin@mfu.ac.th

Correspondence author: Tawatchai.api@mfu.ac.th (TA)

Abstract

Background: One of the major barriers to accessing health care services is experiencing stigma from healthcare workers. Individuals can be greatly impacted by stigma owing to be classed

according to particular characteristics, such as being a tribal member. This study aimed to understand the experiences and adaptations of hill tribe people in Thailand who faced stigma when accessing health care services.

Methods: A phenomenological qualitative method was used to elicit information from hill tribe members who had prior experience in accessing health care. A question guide was used to interview the participants. Interviews were conducted in hill tribe villages in a private and confidential room in August 2021. Each interview lasted for 45 minutes.

Results: A total of 85 people participated in the study: 25 men and 60 women. Akha and Lahu people were the majority. A large proportion had no education and the average monthly income was 2,500 baht per family. Three forms of stigma were detected among the hill tribe people while accessing health care services in different hospitals: verbal stigma, physical stigma, and negative looks. Three levels of impacts were found: completely not understood with no impact, little understanding with little pain, and fully understood with full impact. Two response approaches were identified: nonresponse and response (proper, nonproper immediate response, and assertive response). Three factors were protective against stigma: speaking fluent Thai, wearing modern clothing, and the ability to pay medical fees.

Conclusions: Hill tribe people face several forms of stigma. This results in various levels of impact and different response methods. Some factors can be protective against encountering stigma while accessing health care services in a hospital. Implementing a program to reduce stigma should be focused on these factors to improve the level of access to care among the hill tribe population.

Keywords: Stigma, experiences, hill tribe, access to public health and medical services

Introduction

The American Psychological Association (APA) defines stigma as a negative social attitude attached to a characteristic of an individual; such characteristics may be regarded as a mental, physical, or social deficiency [1]. Stigma occurs when people are perceived as different from other people, particularly those who have specific characteristics, such as those with lower economic [2–4] and educational statuses [5] and living with different cultures and social norms [6, 7]. There are many forms of the stigma presentations such as in verbal [8] and physical presentations [9]. Verbal stigma is the form of voice and language used of ones' negative attitude to the other [10]. While physical stigma could present in various forms of body languages to present ones' negative attitude to the other [10]. The impact can be prominent when one group of people has power over

the other [10, 11]. Stigma is an issue when there is unequal access to fundamental human rights, including access to public health and medical care systems, as reported by Scott et al. [12] and Stangl, et al [13]. The impact of stigma on accessing medical care for those with less power according to their status will cause short- and long-term effects. Stigma is an issue in almost all societies, in both developed and developing countries including Thailand, and is found with different patterns, forms, manifestations and outcomes.

Thailand has been recognized as a successful country owing to its public health and medical services and public health institutes, which extend to the village level [14]. However, many groups of people experience stigma when accessing public health and medical services [15]. This is often due to their lower education and economic status [16-18]. The impact of stigma is compounded by other characteristics, such as for the hill tribe people in Thailand who live along the border of Thailand and Myanmar, with more than 4.5 million residents in 2020 [19]. The hill tribes living in Thailand have their own culture, norms, languages, and lifestyle [20]. Even though Thailand is known for its capacity for its health care services, some minority populations are still not covered by this service. Many communicable childhood diseases and premature deaths have been reported among these populations [15, 21]. These outcomes could be influenced by factors that play a role in reducing access to public health and medical services through governmental health care institutes.

Thailand implemented universal health care in 2002 [14, 22] to ensure that all Thai citizens could access standard and qualified public health and medical services. However, the universal coverage scheme covers only those who hold Thai identification card with 13 digits [22]. While, the basic health care services are provided without charge to all people living in Thailand, with a particular focus on childhood immunizations and maternal and child health care services [23, 24]. However, many health care problems have been frequently reported among hill tribe people [25]. These problems might be caused by stigma from health care providers. Apidechkul 26] reported that Thai HIV/AIDS people had a survival time significant longer than those the hill tribe HIV/AIDS patients. Meanwhile, Tamornpart, et al [27] reported that a large proportion of the hill tribe elderly diabetes could not control their blood glucose after treatment. Therefore, this study aimed to understand the stigma experience and adaptations among hill tribe people when accessing health care services. Most hill tribe people favored to access healthcare services in district hospital and health promoting hospital located in subdistrict level [28]. The findings could

help to understand the phenomena of stigma among the hill tribes and could be used to support modifications of the Thailand health care system, which should be fully provided to these populations.

Methods

A phenomenological qualitative method was used to elicit data from participants who belonged to the hill tribe people living in villages in the Mae Fah Laung and Mae Chan districts, Chiang Rai Province, Thailand. The selected villages are located in border areas of Thailand and Myanmar. The target population was hill tribe people aged ≥15 years who had attended a hospital at least twice within the previous year and experienced stigma while at the hospital.

A set of question guides was developed from a literature review and was tested in preliminary in-depth interviews with 6 key hill tribe informants (3 women and 3 men) who had experienced stigma while accessing health care services. The questions were also assessed for quality by three experts: one hill tribe person who graduated with a university degree in health science, one hill tribe village headman, and one hill tribe village health volunteer.

The final question guide consisted of nine questions: 1) Have you ever experienced stigma while attending a hospital? 2) Could you please give me details of the stigma you experienced? 3) Who caused the stigma against you—a doctor, nurse or someone else? 4) How frequently did you experience stigma? 5) How did you feel while experiencing stigma? 6) How did you respond to the event? 7) Did it impact your health? 8) Why did you not receive good service from the health care provider? 9) What expectations did you have before accessing the health care service? (Additional file 1, Question guide)

After obtaining ethical approval from the Chiang Rai Provincial Public Health Office for conducting research with human subjects, village headmen were contacted, and details of the study, including the objectives, were explained to them. Data were collected from the hill tribe people living in four villages: one village located close to a district hospital, one village located close to a primary health care hospital, and another two villages located far from any city or hospital. People who met the criteria were invited to participate in the study. The interviews were conducted in August 2021 in a private and confidential room in the community hall that was prepared for this purpose. Interviews were conducted by interviewers who had much experience in qualitative research and were familiar with the hill tribe people. Before the formal interviews, the interviewers introduced their background to the participants. Re-interview was used in case of information obtained was not

saturated or confusing or contrasting of the information within and between participants. The interviews lasted 45 minutes each. All interviews were recorded after obtaining approval from the participant, and field notes were taken.

The records were typed and checked for errors before coding. The transcripts were returned to the participants to check the accuracy of the information before further analysis. The saturation of information was discussed among the researchers. Coding trees of the main findings were developed, and key findings were preliminarily extracted. Next, all information was transferred into the NVivo program (NVivo, qualitative data analysis software; QSR International Pty Ltd., version 11, 2015) to derive the themes. The findings were reconsidered by the research members and confirmed by the participants before conclusions were made.

Ethics approval and consent to participate

All research protocols, including the question guide, were approved by the Chiang Rai Provincial Public Health Office (CRPPHO No. 69/2564). All activities were performed in accordance with the relevant guidelines and regulations. Before the interviews, all participants were provided information. Informed consent was obtained on a voluntary basis. Those who could write were asked to provide a fingerprint representing informed consent on a voluntary basis. For those younger than 18 years, informed consent was also obtained from their parents before the interviews. All records were properly destroyed after completion of the analysis. Information from the study was presented anonymously without being able to identify an individual's details.

Results

General characteristics

A total of 85 participants were recruited into the study: 25 men and 60 women. The majority were Lahu (36 people), Akha (34 people), and Thai Yai (11 people). The average age was 44.8 years (min=15, max=70). Sixty people (70.6%) were Christian, 71.85 had never attended a school, 59.8% worked in the agricultural sector, with a median monthly income of 2,500 baht ($84) per family (Table 1).

Table 1 Characteristics of participants

Characteristics	n	%
**Total**	**85**	**100.0**
**Sex**		

Characteristics	n	%
Male	25	29.4
Female	60	70.6
**Age** (years)		
<35	22	25.9
36-50	32	37.6
>50	31	36.5
mean = 44.8, min = 15, max =70		
**Tribe**		
Akha	34	42.4
Lahu	36	40.0
Thai Yai	11	12.9
Lisu	3	3.5
Yao	1	1.2
**Marital status**		
Single	11	12.9
Married	64	75.3
Ever married/ divorce/ separated	10	11.8
**Religion**		
Christian	60	70.6
Buddhist	25	29.4
**Education**		
No education	61	71.7
Primary school	6	7.1
High school	14	16.5
College/University	4	4.7
**Occupation**		
Agriculture	49	57.6
Unemployed	18	21.2
Employee	12	14.2
Merchant	3	3.5
School attending	3	3.5
**Monthly income** (bath) median = 2,500, interquartile range (IQR) = 1,000-3,750		

The analysis identified four types of stigma, three levels of impact, two response approaches, and three factors that acted as a protector against stigma among the hill tribe people while accessing health care services in different hospitals. The majority of the people preferred to access a primary health care hospital, which is a small hospital located at the village level. At this level, all primary care services are available, including family planning and reproductive health, immunizations for children, etc. The second choice for hill tribes was to access medical care at a district hospital, which is the secondary level. A few cases accessed a tertiary hospital located in the city when they had a severe illness.

Forms of stigma

Several forms of the hill tribe people experienced stigma were found, including verbal, physical, and negative looks, particularly from healthcare workers working in secondary hospitals or district hospitals. Nurses were the major contributors to all forms of stigma against the hill tribe people. Four different forms of stigma experience were prevalent among the hill tribe people in Thailand.

a. Verbal

Stigma through verbal language was the major form of stigma, especially for those who could understand Thai at any level. The expression of healthcare workers through language had a large impact on these hill tribe members because they could understand the context of the communication. A large proportion of the hill tribe members could not speak fluent Thai, including having difficulties with the accent while speaking, which was not fully understandable by native Thai speakers. This could lead to poor confidentiality and the patient being afraid of the healthcare workers. This reflects the powerlessness of the patient in relation to the healthcare workers.

A 67-year-old man said [P#10]:

"A few months ago when I visited my doctor to get drugs for hypertension, I saw a bad expression from a nurse. She was angry with me and said that I do not understand what she said. Yes, I did not understand what she said, because my skill in Thai was very poor. However, I thought that as people, we should not say it this way".

A 47-year-old woman said [P#55]:

"When I visited a doctor for a very painful stomach, a nurse asked me to cooperate with her in doing something. However, I had such pain in my stomach that I could not follow her. She then spoke badly to me. Even though I felt a lot of pain, I also did not like what she said. However, I did not respond to her because I feared that I might not receive good treatment from her."

A 65-year-old woman said [P#58]:
"Last month, I went to a hospital to get drugs, and I saw a nurse speaking harshly to an old Akha lady. She said something that the old lady did not understand, and she spoke a lot with blaming words. I really didn't like it".

A 47-year-old woman said [P#60]:
"Just last week, while I visited my doctor at a hospital, I was shouted at by a nurse. She was very angry and said that "You are the highland people, why don't you understand what I say?", and it is true that I did not clearly understand what she said".

Being spoken to negatively by a nurse, particularly for those who are older and not fluent in Thai, is a very common stigma experience among hill tribe people in Thailand. Negative verbal expressions had the greatest impact on the emotions of the hill tribe people while attending a hospital. However, the patients did not respond to the health care workers because they were afraid they would not receive good treatment.

b. Physical

Several physical actions presented as stigma from healthcare workers were reported by the participants, such as throwing paper, finger-pointing, not being spoken to and being ignored, or being the recipient of threatening actions. Almost all forms of the stigma were made by healthcare workers who were working at district hospitals. These physical actions were experienced by all of the participants who neither spoke nor understood Thai. This type of stigma was most frequently reported as occurring in the outpatient and labor departments. Some women reported having been physically abused during labor.

A 16-year-old man said [P#25]:

“Maybe 4–5 months ago, I got in a traffic accident and needed to have my wounds dressed at a hospital. The nurse who treated me processed me very roughly, which hurt. I saw that she treated me without special care. I felt that she was just practicing wound dressing on me because she knew that I was a hill tribe member. Before dressing my wound, she called my name and asked “Are you from a hill tribe?”.

A 64-year-old man said [P#73]:

“This was very important for my life. I went to see a doctor to get drugs for HT. I was diagnosed with HT several years ago. On the day, I left my home very early. I sat down close to a nurse when I reached the hospital. Immediately, the nurse moved to another place. I know that sometimes this happens, but in my case I felt that the nurse did not like me and did not want to sit close to me. Yes, absolutely, I accepted it”.

A 24-year-old man said [P#65]:

“A couple of years ago I had dengue fever, and unfortunately I was admitted to the hospital. During admission, I was cared for less than another patient whose bed was beside mine at the hospital. The nurse had many conversations with that person, but she did not even look at me. I felt that I had a very high fever and I needed someone to help me. I requested medicine from her, but she did not respond. I know that she might have a lot of work to do, but she should fairly care for everyone the same.”

Physical stigma was another important type of stigma experienced by hill tribe people while attending a hospital. Physical stigma occurred during under the medical procedures and in the documentary process.

c. Negative looks

In Thai culture, cynical or negative looks are another form of stigma that has a great impact on the victim. Thai women frequently engage in this type of stigma. It means that someone looks bad or is not equal in some aspect, particularly regarding economic and educational status. Stigma from negative looks can impact anyone, without the need for understanding the verbal language. This

form of stigma does not only occur between Thai health care workers and hill tribe patients. It is also common in daily life between a person who has a higher economic or educational status than another.

A 53-year-old woman said [P#50]:
“I clearly remember when I attended a hospital last month. I do not have an ID card and I did not attend school. Therefore, I was not confident to talk about my symptoms with the nurse because I was afraid that I would not speak well and make her understand my points. However, when I made eye contact with the nurse, all my words disappeared. My feeling at that time was that I was afraid to say anything to her because she looked at me so unfriendly”.

A 47-year-old man said [P#54]:
“One day I visited a doctor, and before meeting with them a nurse called me in to take my history. During that time, I felt that I was a very little man because she looked at me harshly and said “Do you have money to pay for doctor fees?” I spoke very little and said unconfidently that I had little money. With the way she looked at me and talked to me, do you know it was very upsetting.”

A 49-year-old woman said [P#53]:
“This was a very particular experience to me. Maybe two months ago, when I visited a doctor, a local Thai woman looked at me from head to toe. I felt that she did not like me. I could sense that she looked at me like a poor person. I did not like that situation.”

Stigma presented as negative looks impacts many people, including hill tribe members. This form of stigma can be easily inflicted by both health care workers and the general Thai public. Negative looks can discourage people, making people think of themselves in negative ways, especially hill tribe people who live in poor communities and tribes in Thailand.

Level of the impact

a. Completely not understood with no impact

At this level, there was no impact on the hill tribe individual because they did not understand the meaning of the speaker. However, the impact was detected by their relatives who cared for the patient while at the hospital. Therefore, when asking the participant about their experience of

stigma, they responded that there was no impact; however, impacts were felt by their relatives who took them to the hospital. This means that individuals who could not speak Thai would not be directly impacted by the stigma, particularly in verbal form, but it would impact their relatives.

A 44-year-old woman said [P#51]:
"When I visited the doctor, I had many words from the nurse but did not understand what she said. At that time, I felt nothing. However, after coming back home, my son told me that the nurse spoke to me very roughly and repeated it over and over! However, anyway, I do not know and I do not want to know what she said."

A 60-year-old woman said [P#80]:
"A year ago, one of my nephews brought me to the hospital, and I was admitted. During the hospital stay, my nephew told me that a nurse said very hard words to me, including saying that I had a bad smell and why didn't I take a shower?"

She added
"Luckily, I did not know about what she said, and I felt nothing. Is that good?" (laughing).

A 44-year-old man said [P#49]:
"I guess that I had many hard words from nurses while attending the hospital. However, I did not understand Thai, so I did not know the meaning. However, my son said that the nurses were rough to me. However, I did not feel bad anymore".

b. Little understanding with little pain

Some hill tribe people could speak Thai, and they understood the meaning of what the health care worker said, which made them feel bad. The majority of the conflict occurred while the patient tried to explain what they wanted or needed help with, but their key ideas were not completely understood by the health care worker. The level or respect of the approach from the health healthcare workers to the clients were completely proper or enough or direct to the need, this was the origin of the pain. Commonly, people accessing medical care urgently need help to relieve their suffering, and effective communication is crucial between the patients and health care workers. Without effective communication, misunderstandings could impact several things.

A 65-year-old woman said [P#58]:

“Basically, I can use Thai a little. One day last year I went to see the doctor for strong abdominal pain. I told the nurse about my problem, but she did not understand what I said. Moreover, I knew that she complained about me, but I did not clearly understand. The next day, my son told me that the nurse said very hard words to me, but I felt nothing because I did not understand what she said.”

A 47-year-old woman said [P#57]:

“Last year I met a nurse while attending a hospital to get medicine for my hypertension illness. She told me something very fast in Thai. After a while she asked me, “Did you understand what I said?” I nodded my head to show her I understood. In fact, I got little information from her. Do you know why I did that—because I was afraid she would not care for me properly.”

c. Fully understood with full impact

Stigma can cause hurt and pain in anyone. It will increase the severity while it sends a completely negative message between people. Because they fully understood the negative stigma from health care workers, the hill tribe patients experienced hurt and pain. Almost all hill tribe people less than 30 years old speak fluent Thai, and this particular group was impacted by fully understanding the negative words of the health care workers.

A 16-year-old woman said [P#25]:

“Last year I was referred to the emergency room due to a car accident. It was almost 11:30 pm. The nurse did the wound dressing very roughly, which hurt a lot. During this, she swore that the hill tribe people never use motorcycles carefully, which is not true. The accident was caused by heavy rain. I felt that she did this because we are from the hill tribe.”

A 28-year-old woman said: [P#23]:

“Last week I visited the hospital. One of the staff members asked me “Do you have a Thai ID card?” I felt that this question had the same meaning as “Do you have money?” She asked me

other questions without looking at me such as “Which mountains are you from?” I felt very bad and shy.”

A 30-year-old man said [P#42]:
“Two years ago I brought my father to the hospital. I heard a nurse ask questions to a woman close to me who was a hill tribe member and could not understand Thai. The nurse said that “Most of the hill tribe people do not understand anything. When will they pass away?” I looked at her, and I felt very angry even though she did not talk with me directly.”

The impacts of stigma depended on when people could use and understand Thai. Those who could understand only a little had little impact from the stigma because they could not understand the meaning of the words. The fear of being improperly cared for was another concern among the hill tribes facing stigma from health care providers.

Responses

Several forms of the response were observed by the hill tribe people when they experienced stigma from health care workers. A nonresponse was one of the responses when the patient did not say anything. This did not necessarily mean that they did not feel stigma, but that they felt they had no choice about responding, which was deeply hurtful. A nonresponse indicated that there was no equity between the two people. Some people kept silent because they were afraid that they would not receive good care from the health care worker. Other people chose to respond in proper or improper ways, including aggressively.

a. Nonresponse

There were two reasons for a nonresponse to stigma. First, the recipient understood everything but kept silent out of fear. They feared that they would not receive good quality care from the health care worker. This is the result of unequal power, especially concerning the power to save a life. The second reason was that the patient did not understand the context of what was said in Thai. Therefore, they did not respond to the situation. A nonresponse due to not understanding the context commonly occurred among the elderly population. Owing to a limited

use of Thai and a different culture, they did not understand the basic aggressive words and negative physical language.

A 35-year-old woman said [P#33]:
"A year ago I brought my mom to visit the hospital. A nurse spoke to my mom and me harshly, and she blamed us poor people who could not afford to pay for the treatment. However, we did not respond to her. We kept quiet because we were afraid that she may not take care of us properly."

A 47-year-old woman said [P#55]:
"Five years ago I got a hand injury while working on the farm. I went to see a doctor at a hospital. Before meeting the doctor, I had been asked many questions by a nurse in the emergency room. Some questions were truly hurtful to me, but I did not respond because I feared I would not be cared for properly."

A 42-year-old woman said [P#56]:
"Last year I went to see the doctor about my diabetes mellitus. One of the staff told me to fill out a form. I told him that I could not write. Immediately, he shouted at me with some bad words. I was sitting close to him, and I did not know I should do the next step. I kept everything in my heart because I feared that he would not take care of me."

b. Response

Responses to bad situations including conversations and other interactions between hill tribe patients and health care workers were present at three levels: proper response, nonproper immediate response, and aggressive response. Responses occurred among the hill tribe members with fluent Thai and aged 35 years and below. In this age group, almost all had attended school at some level and were exposed to people outside their village, including familiarity with other cultures and lifestyles. Because they were frequently exposed to negative messages and actions, these patients preferred to respond when they experienced negative messages or improper actions from the health care workers.

Proper response: In this type of response, the patient responds calmly and directly when a problem arises. Most responses were initiated by relatives; however, this situation indicates the lower power or understanding of the patient.

A 23-year-old man said [P#9]:
"I remember that one day in 2017, I visited a hospital with a severe headache. A nurse called my name and asked me for my history and checked my blood pressure. The nurse asked me to explain my problem. I had only said a few words when she shouted "Go wait there". I kept everything normal and responded "I would very much appreciate it if you told me with normal words. I understand how busy you are today." and I smiled at her."

A 30-year-old man said [P#10]:
"Last year I brought my wife to see a doctor at a primary care hospital to check her health during pregnancy; this was a small health center located in our village. The staff member asked my wife questions, and I responded that she could not speak Thai. The staff member turned to my wife and asked why she could not speak Thai. I was slightly surprised and responded "How many languages do you speak because of graduating with a university degree? She never attended school—why do you expect her to speak Thai?"

Nonproper immediate response: In this response type, the primary responders were patients. When they were already suffering, receiving stigma from health care workers was frequently experienced by hill tribe people who became sick and needed to access health care services.

A 47-year-old man said [P#54]:
"Last month when I visited the diabetes mellitus clinic at a hospital, the staff member shouted at me with hard words. I looked at her face immediately and then stood up. After that, she calmed down and talked to me normally."

Assertive response: Some people reported aggressive responses. The major reason for such a response was a long wait time or ignoring severe suffering. Aggressive responses were frequently

reported in outpatient departments and labor rooms when patients were severely suffering but did not receive proper care.

A 20-year-old woman said [P#76]:

"Last year my son who was five years old got a foot injury, and I brought him to the hospital emergency room. A nurse cleaned his wound very roughly, and my son told me that it hurt a lot. I felt that she did not treat him carefully and mindfully. I immediately told her "Could you please treat my son better, as he is a human? I felt very angry."

A 65-year-old man said [P#65]:

"One day a couple of weeks ago I visited a hospital because I had a very bad headache. I waited in a long queue. A few moments later, a man who was dressed in a very modern style came and jumped the queue in front of me, and the nurse said it's fine. I felt that it was not ok, and I asked the nurse to explain what happened. I started to ask many people who were sitting down in that area."

Many hill tribe people chose not to respond to the stigma because they feared being improperly cared for. When people did respond, there were three response forms: proper, nonproper, and aggressive. The responses were dependent on the situation and on whether the patient was a loved one, such as a son.

Protectors

While stigma was commonly encountered by the hill tribe people, there were at least three factors that were protective against stigma in the context of accessing health care service: having money to pay for services, wearing modern clothing, and speaking fluent Thai.

a. Speaking fluent Thai

Being able to speak fluent Thai was protective against stigma in any form received from health care workers. The ability of the hill tribe patients to communicate smoothly and effectively in Thai hill reduced the incidence of stigma and its negative effects. The fluent use of Thai increased the respect from health care workers in all of the mentioned health care departments.

A 26-year-old man said [P#82]:

"I am an Akha and I graduated high school. I can speak Thai very well. I know that some people look physically different, and that cannot be changed. However, we can adapt ourselves. When I go to a hospital, I can speak Thai, then I never have a problem with them. Speaking Thai truly helps me."

A 24-year-old man said [P#63]:

"A couple of years ago I visited a hospital to seek care after getting in a traffic accident. At the emergency room, I received good treatment. The nurse was very polite to me. This was because I could speak with them very well, even though I am Akha."

A 49-year-old woman said [P#84]:

"I am Lahu, and I have lived in Thailand for 20 years. I did not attend school, but I can speak Thai because of my daily practice with Thais. I have not had any problems while attending hospitals."

b. Wearing modern clothing

Hill tribe people wearing their traditional clothes were more likely to encounter stigma or have a negative experience with health care workers. When the hill tribe people wore modern clothing, they experienced less stigma. Stigma could be completely absent if they wore modern clothes and spoke fluent Thai. However, most hill tribe women in all tribes commonly wear their traditional style, which is part of their social culture. Thus, hill tribe women faced more frequent and severe stigma when accessing health care services. Women who wore their traditional clothing and could not speak fluent Thai faced more severe stigma from health care workers.

A 47-year-old woman said [P#57]:

"Actually, the hill tribe people always dress in the traditional form. However, I prefer to dress in modern clothes when attending a hospital. I feel that I receive more respect from people, including health care staff, while wearing modern clothes."

A 20-year-old woman said [P#61]:

"I am Akha, I have noticed unfriendly looks from people around me. I decided to wear modern clothes, and I was surprised that I had less negative attention from people around me when I went to the city and hospital."

c. Having money for medical fees

Most hill tribe people who could afford medical fees preferred to attend a private hospital. In a private hospital, the services are based on a mix between standard health professional practice and private service. Anyone who accesses a private hospital will be served better than at public hospitals, which are nonprofit organizations. Hill tribe members who can afford it will choose private hospitals to reduce stigma. Furthermore, the service is always better and quicker than at a public hospital.

A 43-year-old woman said [P#39]:
"Three years ago I felt very bad while visiting a hospital. I was asked for money to pay the medical fees before receiving care. I told them that I had a lot of money, and the actions from the hospital staff changed immediately. They were nice to me and I had good care from them. It's not right! "

A 28-year-old woman said [P#23]:
"I prefer to go to a private hospital. Although we have to pay medical fees, we will receive good care. In a public hospital, I do not need to pay all the medical fees because I have a Thai ID card, but I feel that I have not had proper care from them. My friends say that money is very important to get good service from a hospital."

Those who dressed in modern clothing, spoke Thai well, and could afford to pay medical fees experienced less stigma. These characteristics indicate a higher level of socioeconomic status in the social environment. Those who had a lower socioeconomic status could suffer in two ways—from their lower status and from experiencing stigma.

Discussion

The hill tribes living in northern Thailand suffer from several forms of stigma when accessing health care services; these include verbal and physical stigma and negative looks. Different levels of impact from stigma were identified when the tribe members accessed health care services, which were

dependent on understanding Thai: completely not understood with no impact, little understanding with little pain, and fully understood with full impact. The hill tribe members had two main approaches when responding to stigma: non-response and response (proper, nonproper immediate response, and aggressive response). Some characteristics were found to be protective against stigma: speaking fluent Thai, wearing modern clothing, and the ability to pay medical fees.

The hill tribe people faced several forms of stigma while attending a hospital, such as verbal sigma, physical stigma, and negative looks. The socioeconomic conditions of the hill tribe members are different from those of health care providers, who are primarily of the Thai majority. The hill tribe members detected several forms of stigma, and some people reported experiencing more than one form while in a health care setting. Verbal stigma from health care workers was the primary form that caused a large amount of suffering among the hill tribe members. The major cause of verbal stigma was the insufficient understanding of Thai by the hill tribe people when health care workers tried to give information to the patients. The hill tribe patients were unable to understand what was being said; this was the main source of the problem. Physical stigma occurred when there was a required interaction between a health care provider and the patient, especially when it required the cooperation of the patient during a medical procedure. Unfortunately, the hill tribe patients could not understand what was being said, which caused problems. Stigma enacted through negative looks resulted in a deep perception by the hill tribe members that they were not in the same class as the health care providers. Negative looks express a sense of looking down on another person. This was also found in an Indonesian study that reported that the social and moral perceptions of health care workers impacted their patients through some form of stigma, especially those who had a lower socioeconomic status and severe disease, such as HIV/AIDS [29]. Nyblade et al. [30] reported that stigma in health care facilities was the main cause of death and unsuccessful treatment, particularly for several chronic diseases, such as HIV/AIDS and tuberculosis, and especially for those who lived under socioeconomic constraints. Another study confirmed that verbal stigma could cause individuals to suffer and reduced their self-esteem, which could have several consequences throughout the lifespan, especially for those in vulnerable populations [31]. Livingston [32] reported that stigma was a primary source of mental health problems, particularly for those who were poor, were substance users, or part of a minority population. Qualitative studies in Thailand reported that some groups of people faced severe stigma while in various settings, including within their own families and workplaces [33]. However, there is no evidence of stigma among the hill tribes

themselves while accessing health services, particularly regarding physical stigma and negative looks.

The major influencer of stigma propagated by Thai health care workers towards hill tribe patients is the language barrier. Therefore, the level of impact on the hill tribe people was classified according to their ability to use Thai. If a person could not speak Thai, they had no understanding of the negative words of the health care worker, in which case they did not detect stigma. Conversely, those who completely understood what was said in Thai fully suffered from the stigma experience. Earnshaw et al. [34] reported that receiving stigma from health care workers was strongly associated with poor health and health care quality, especially for those living with chronic disease. Moreover, Knaak et al. [35] reported that stigma in the health care setting had both direct and indirect impacts on multiple levels for patients owing to the higher socioeconomic standing of health care workers, resulting in a decreased health status of patients. A report from Thailand demonstrated that stigma was a major cause of poor treatment outcomes among HIV/AIDS patients and that promoting a good organizational culture and developing health care workers' interpersonal skills could minimize the impact of stigma on patients [36].

Owing to the specific characteristics of the hill tribe people in Thailand, some preferred not to respond to avoid getting poor care. Others responded with either a proper approach or a nonproper approach, including aggressive responses to the situation. Those who preferred to respond improperly or aggressively were overcome by their emotions. This might be the result of repeatedly suffering from stigma. After conducting a literature search of several data sources, no reports were identified that investigated the response to stigma, particularly in the health care setting. Owing to the long-lasting experiences of stigma, as well as suffering from their medical condition, some patients preferred to respond. Many hill tribe members preferred to limit their response because they were afraid of not being treated properly by health care workers. An aggressive response was reported by a patient fluent in Thai who was upset by the negative words of a health care worker. This response was from a person who was socioeconomically equal to the healthcare workers. This indicates that if hill tribe people had a higher socioeconomic status, they would more often voice their opinions.

In our study, three characteristics of the hill tribe people were identified as protective factors against stigma from health care workers: the ability to speak Thai fluently, wearing modern clothing, and having money to pay for medical fees. These characteristics particularly protected the hill tribe

people from stigma encountered when attending a hospital. However, some of these characteristics are very difficult to improve or adopt, particularly being able to speak Thai fluently and having money to pay for medical fees. The adaptability decreased among hill tribe members over 40 years of age because they never attended school and typically lived in poor families. However, younger people aged less than 30 years have more opportunities to reduce stigma and its impact because they attend school, are able to speak fluent Thai, and often have better jobs. Although there are no previous studies to support our findings within the specific context of this population, our findings indicate that people can adapt to avoid or reduce stigma in their lives.

This study has some limitations. Some participants may have been reluctant to tell the researchers about their negative experiences when accessing health care services because they were afraid of it having a negative impact the next time they needed to go to the hospital. The researchers took time to explain that participating in the study would not negatively impact them and ensured them that their information would not be released and that they would not be individually identified. Regarding this issue, it is very important to have clear processes in a study, including maintaining the confidentiality of the information obtained. Some of the information obtained from participants was conflated with bad feelings about their experiences while thinking back on it, which required the researchers to make a determination before coming to conclusions. Compared with male interviewers, female interviewers were often more familiar to the participants, allowing them to better express their suffering. Therefore, researchers could assess whether the findings had become saturated by re-interviewing participants with a new female interviewer. This ensured that maximized and saturated information was obtained from participants.

Conclusion

Three forms of stigma were detected among the hill tribe people while accessing health care services in different hospitals: verbal stigma, physical stigma, and negative looks. Three levels of impacts were found: completely not understanding with no impact, little understanding with little pain, and fully understood with full impact. Two response approaches were identified: nonresponse and response (proper, nonproper immediate response, and aggressive response). Three factors were found to be protective against stigma: speaking fluent Thai, wearing modern clothing, and the ability to pay medical fees. All healthcare workers who are working or who are assigned to work in the hill tribe villages should be trained specific program to improve knowledge and attitude in working with

people who have different background. Provincial public health office should develop a monitoring system to ensure that all people will be cared without the stigma. Training the basic hill tribe languages to healthcare workers who will be assigned to work in the hill tribe villages are also seriously recommend to avoid the stigma from language barriers.

Supplementary information

S1 Appendix. Question guide.

Acknowledgments

We thank Mae Fah Luang University and The Center of Excellence for The Hill Tribe Health Research for supporting the grants. We are also grateful for all health care workers who worked in the study setting for their assistance in obtaining access and recruiting participants. Finally, we thank all of the participants for their participation in the study.

Author Contributions

Conceptualization: Peeradone Srichan and Tawatchai Apidechkul

Data curation: Peeradone Srichan, Tawatchai Apidechkul, Ratipark Tamornpark, Thanatchaporn Mulikaburt, Pilasinee Wongnuch, Siwarak Kitchanapaibul, Panupong Upala, Chalitar Chomchoei, Fartima Yeemard, Anusorn Udplong, Onnalin Singkhorn

Formal analysis: Peeradone Srichan, Tawatchai Apidechkul, Ratipark Tamornpark, Thanatchaporn Mulikaburt, Pilasinee Wongnuch, Siwarak Kitchanapaibul, Panupong Upala, Chalitar Chomchoei, Fartima Yeemard, Anusorn Udplong, Onnalin Singkhorn

Funding acquisition: Tawatchai Apidechkul

Investigation: Peeradone Srichan, Tawatchai Apidechkul, Ratipark Tamornpark, Thanatchaporn Mulikaburt, Pilasinee Wongnuch, Siwarak Kitchanapaibul, Panupong Upala, Chalitar Chomchoei, Fartima Yeemard, Anusorn Udplong, Onnalin Singkhorn

Methodology: Peeradone Srichan and Tawatchai Apidechkul

Project administration: Tawatchai Apidechkul

Writing—original draft: Peeradone Srichan, Tawatchai Apidechkul, Ratipark Tamornpark, Thanatchaporn Mulikaburt, Pilasinee Wongnuch, Siwarak Kitchanapaibul, Panupong Upala, Chalitar Chomchoei, Fartima Yeemard, Anusorn Udplong, Onnalin Singkhorn

Writing—review & editing: Peeradone Srichan, Tawatchai Apidechkul, Ratipark Tamornpark,

Thanatchaporn Mulikaburt, Pilasinee Wongnuch, Siwarak Kitchanapaibul, Panupong Upala, Chalitar Chomchoei, Fartima Yeemard, Anusorn Udplong, Onnalin Singkhorn

Competing interests

The authors have declared that no competing interests exist.

References

1. American Psychologic Association (APA). Stigma, prejudice and discrimination against people with mental illness. Available from: https://www.psychiatry.org/patients-families/stigma-and-discrimination

2. Makleff S, Wilkins R, Wachsmann H, Gupta D, Wachira M, Bunde W, et al. Exploring stigma and social norms in women's abortion experiences and their expectations of care. Sex Reprod Health Matters. 2019; 27(3):50–64. DOI:10.1080/26410397.2019.1661753.

3. Incollingo AC, Smieszek SM, Nippert KE, Tomiyama AJ. Pregnant and postpartum women's experiences of weight stigma in healthcare. BMC Pregnancy Childbirth. 2020; 20(1):1-10 DOI:10.1186/s12884-020-03202-5.

4. Medina PL, Scott J, Chapman S, Barnett J, Dack C, Family H. A qualitative study on intersectional stigma and sexual health among women on opioid substitution treatment in England: Implications for research, policy and practice. Soc Sci Med. 2019; 222: 315-22. DOI: 10.1016/j.socscimed.2019.01.022.

5. Seidu AA, Agbaglo E, Dadzie LK, Ahinkorah BO, Ameyaw EK, Tetteh JK. Individual and contextual factors associated with barriers to accessing healthcare among women in Papua New Guinea: insights from a nationwide demographic and health survey. Int Health. 2020;0:1-13 DOI: 10.1093/inthealth/ihaa097.

6. Keller S, McNeill V, Honea J, Paulson Miller L. A look at culture and stigma of suicide: textual analysis of community theatre performances. Int J Environ Res Public Health. 2019;16(3):1-20. DOI: 10.3390/ijerph16030352.

7. Ran MS, Hall BJ, Su TT, Prawira B, Breth-Petersen M, Li XH. Stigma of mental illness and cultural factors in Pacific Rim region: a systematic review. BMC Psychiatry. 2021;21(1):1-16. DOI: 10.1186/s12888-020-02991-5.

8. Wang L. Verbal stigmatization from family: how does that affect Latino men who have sex with men. Journal of Social and Personal Relationships. 2019; 36(1): 3021.

9. Mazurkiewicz N, Krefta J, Liowska M. Attitudes towards appearance and body-related stigma among young women with obesity and psoriasis. Frontiers in Psychiatry. 2021; 12: 1-11. DOI: 10.3389/fpsyt.2021.788439.
10. Xu M, Macrynikola N, Waseem M, Miranda R. Racial and ethnic differences in bullying: review and implications for intervention. Aggress Violent Behav. 2020; 50: 1-45 DOI: 10.1016/j.avb.2019.101340.
11. Kontos P, Radnofsky ML, Fehr P, Belleville MR, Bottenberg F, Fridley M, et al. Separate and unequal: a time to reimagine dementia. J Alzheimers Dis. 2021;80(4):1395-99. DOI: 10.3233/JAD-210057.
12. Scott N, Crane M, Lafontaine M, Seale H, Currow D. Stigma as a barrier to diagnosis of lung cancer: patient and general practitioner perspectives. Prim Health Care Res Dev. 2015;16(06):618–22
13. Stangl AL, Earnshaw VA, Logie CH, Brakel W v, Simbayi LC, Barrie I, Dovidio JF. The health stigma and discrimination framework: a global, crosscutting framework to inform research, intervention development, and policy on health-related stigmas. BMC Medicine. 2019; 17(31) 1-13. DOI: 10.1186/s12916-019-1271-3.
14. Tangcharoensathien V, Tisayaticom K, Suphanchaimat R, Vongmongkol V, Viriyathorn S, Limwattananon S. Financial risk protection of Thailand's universal health coverage: results from series of national household surveys between 1996 and 2015. Int J Equity Health. 2020;19(1):1-12. DOI: 10.1186/s12939-020-01273-6.
15. Apidechkul T. A 20-year retrospective cohort study of TB infection among the Hill-tribe HIV/AIDS populations, Thailand. BMC Infect Dis. 2016;16:1-14. DOI: 10.1186/s12879-016-1407-4.
16. Chomchoei C, Apidechkul T, Wongnuch P, Tamornpark R, Upala P, Nongkhai MPN. Perceived factors influencing the initiation of methamphetamine use among Akha and Lahu youths: a qualitative approach. BMC Public Health. 2019;19(1):1-11DOI: 10.1186/s12889-019-7226-y.
17. Chomchoei C, Yeemard F, Tamornpark R, Upala P. Burden and factors associated with hypertension among the adult population of the Lisu Hill tribe, Northern Thailand. J Health Sci Altern Med. 2021;3(1), 9-15.

18. Chomchoei C, Apidechkul T, Khunthason S, Kullawong N, Keawdounglek V, Tamornpark R, et al. Prevalence and factors associated with type 2 diabetes mellitus among middle-aged and elderly Lisu hill tribe people, northern Thailand: a cross-sectional study. J Health Sci Altern Med. 2019;1(3):17-23.
19. World Health Organization (WHO). Regional office for south-east, Asia. (2017). WHO country cooperation strategy, Thailand: 2017–2021. Available from: https://apps.who.int/iris/handle/10665/255510 Assessed 31 Jan2021.
20. Apidechkul T, Yeemard F, Chomchoei C, Upala P, Tamornpark R. Epidemiology of thalassemia among the hill tribe population in Thailand. PLosOne. 2021;16(2): 1-6.
21. Apidechkul T. Seroprevalence and factors associated with hepatitis B virus infection among the hill tribe youths, northern Thailand. BMC Infect Dis. 2019;19(1):1-11. DOI: 10.1186/s12879-019-3747-3.
22. Kantamaturapoj K, Marshall AI, Chotchoungchatchai S, Kiewnin K, Patcharanarumol W, Tangcharoensathien V. Performance of Thailand's universal health coverage scheme: Evaluating the effectiveness of annual public hearings. Health Expect. 2020; 23(6):1594-602. DOI: 10.1111/hex.13142.
23. Paek SC, Meemon N, Wan TT. Thailand's universal coverage scheme and its impact on health-seeking behavior. Springerplus. 2016;5(1):1-16. DOI: 10.1186/s40064-016-3665-4.
24. Limwattananon S, Tangcharoensathien V, Prakongsai P. Equity in maternal and child health in Thailand. Bull World Health Organ. 2010;88(6):420-7. DOI:10.2471/BLT.09.068791
25. Upala P, Apidechkul T, Suttana W, Kullawong N, Tamornpark R, Inta C. Molecular epidemiology and clinical features of hand, foot and mouth disease in northern Thailand in 2016: a prospective cohort study. BMC Infect Dis. 2018;18(1):1-14. DOI: 10.1186/s12879-018-3560-4.
26. Apidechkul T. HIV/AIDS survival rate among hill tribe population in northern Thailand. Siriraj Med J. 2011; 63: 200-2004.
27. Tamornpark R, Apidechkul T, Upala P, Inta C. Factors associated with type 2 diabetes mellitus among the elderly hill tribe population in Thailand. Southeast Asian J Trop Med Public Health. 2017; 48(5): 1072-82.
28. Apidechkul T, Wongnuch P, Sittisarn S, Ruanjai T. Health situation of Akha hill tribe in Chiang Rai Province, Thailand. Journal of Public Health and Development. 2016; 14(1): 77-97.

29. Fauk NK, Hawke K, Mwanri L, Ward PR. Stigma and discrimination towards people living with HIV in the context of families, communities, and healthcare settings: A qualitative study in Indonesia. International Journal of Environmental Research and Public Health. 2021; 18(5424): 1-17. DOI: 10.3390/ijerph18105424.

30. Nyblade L, Stockton MA, Giger K, Bond V, Ekstrand ML, lean RM, et al. Stigma in health facilities: why it matters and how we can change it. BMC Medicine. 2019; 17(25). 1-15. DOI: 10.1186/s12916-019-1256-2.

31. Wang L. Verbal stigmatization from family: how does that affect Latino men who have sex with men?. Journal of Social and Personal Relationships. 2019; 36(1): 3-21.

32. Livingston JD. Structural stigma in health-care contexts for people with mental health and substance use issues: a literature review. Available from: https://www.mentalhealthcommission.ca/sites/default/files/2020-07/structural_stigma_in_healthcare_eng.pdf

33. Laphon C, Chuemchit M. Social stigmatization access to services and services satisfaction among transgender persons at Thai Red Cross AIDS Research Center-Tangerine project: A qualitative study. J Health Res. 2017; 31(S2): S195-201.

34. Earnshaw VA, Quinn DM. The impact of stigma in healthcare on people living with chronic illnesses. Journal of Health Psychology. 2011; 17(2): 157-168.\

35. Knaak S, Mantler E, Szeto A. Mental illness-related stigma in healthcare: barriers to access and acre and evidence-based solutions. Healthcare Management Forum. 2017; 30(2): 111-6.

36. Siraprapasiri T, Srithanaviboonchai K, Chariyalertsak S, Chuenyen J, Khemngern P, Wongthip N, et al. Stigma and discrimination among health care providers and people living with HIV in health care settings in Thailand: a comparison of finding from 2014-2015 and 2017. Available from: https://hivhub.ddc.moph.go.th/Download/Report/S_D/2_UNAIDS_Final_S_D_Final_Health_care_settings_Comparison_a5.pdf

Question guide

Click here to access/download
Supporting Information
Question guide.docx
