# Supplementary material for: Stigma experiences and adaptations in accessing healthcare services among hill tribes in Thailand: A qualitative study
Source: PLoS One. 2025 May 2;20(5):e0321119. doi: 10.1371/journal.pone.0321119 (PMC12047764; doi:10.1371/journal.pone.0321119)
Supplement: Attachment — Submitted filename: Response to reviewers_peeradone.pdf [file pone.0321119.s005.pdf]

Response to reviewers

Dear Editor,

Thank you very much for allowing us to revise this paper with great comments from reviewers. We are also sorry for the delay in the submission of our revised version because the corresponding author was detected by COVID-19 twice while revising the manuscript, including moving to the United States to study at Harvard Medical School, please accept our apologies for the delay in resubmitting the manuscript. After re-analysis of the data, we seriously revised and imrpoved the whole manuscript, particularly the introduction, methods, and discussion sections including change references. We do very much hope that you are happy with this version.

Thank you,
TK

Editor's comments

Thank you for submitting your manuscript to PLOS ONE. After careful consideration, we feel that it has merit but does not fully meet PLOS ONE's publication criteria as it currently stands. Therefore, we invite you to submit a revised version of the manuscript that addresses the points raised during the review process.

In submitting your revised manuscripts kindly pay close attention to the following areas of concern:

1. Substantially revise your introduction to provide the necessary background for your study, including clarification on different types of stigma and clarify distinctions between stigma from discrimination, supported by appropriate references.

: Thank you so much for the comment, we have seriously revised the whole sections of the manuscript particularly in interdiction, method and discussion according to the comments of reviewer 1 (including several excellent pieces comments presented in PDF file) and reviewer 2.

2. Please revise your "materials and methods" section to clarify your sampling frame, as well as inclusion and exclusion criteria. Also clarify issues of participant selection and methods applied to minimize bias in your study.

: Thank you, this section has been strongly reviewed and revised.

3. Please revise your "results" section to clarify major themes and subthemes. Also describe how data saturation was achieved, and revise Table 1 as recommended by reviewers to exclude/correct inappropriate columns and unnecessary inclusion within your Tables.

: We have re-analysis the whole data set and several pieces have been confirmed, while many other findings have been revised and improved including the correction of the number of “tribe (Akha and Lanu)” in table 1, thank you so much

4. Revise your discussion section with comparative analysis to similar studies in the literature, with appropriate referencing and how these other studies compare with findings from your own study.

: Thank you, this section has been largely revised and improved including moving into the proper place or alignment.

5. Clarify your Conclusions as well as your recommendations.

: We have revised the conclusion, but we would like to confirm the redocumentations under specific context of our location.

6. In addition, substantially revise your entire manuscript to address all grammatical and typographical errors. Consultation with an English language editing service is recommended to address this issue if necessary or if required by the authors.

: Basically, the manuscript has been improved by the AJE twice but definitely, the quality is still very poor. We are so sorry about this and have made our best capacity before submitting this version. If you have any better choice, please kindly let us know.

7. Kindly correct and edit to your COREQ checklist to include page numbers with reference to the manuscript.

: Thank you, it’s revised.

8. Please address all other relevant comments as annotated on the manuscript by reviewer #1 where necessary.

: Thank you, we have revised all points including comments presented in the PDF.

Additional requirements

1. Please ensure that your manuscript meets PLOS ONE's style requirements, including those for file naming. The PLOS ONE style templates can be found at

https://journals.plos.org/plosone/s/file?id=wjVg/PLOSOne_formatting_sample_main_body.pdf and

https://journals.plos.org/plosone/s/file?id=ba62/PLOSOne_formatting_sample_title_authors_affiliations.pdf

2. Please provide additional details regarding participant consent. In the ethics statement in the Methods and online submission information, please ensure that you have specified what type you obtained (for instance, written or verbal, and if verbal, how it was documented and witnessed). If your study included minors, state whether you obtained consent from parents or guardians. If the need for consent was waived by the ethics committee, please include this information.

3. We note that the grant information you provided in the 'Funding Information' and 'Financial Disclosure' sections do not match.

When you resubmit, please ensure that you provide the correct grant numbers for the awards you received for your study in the 'Funding Information' section.

: Thank you, we have added the correct information in the cover letter, please kindly see in cover letter.

4. Thank you for stating the following financial disclosure:

"The funders had no role in study design, data collection and analysis, decision to publish, or preparation of the manuscript."

At this time, please address the following queries:

a) Please clarify the sources of funding (financial or material support) for your study. List the grants or organizations that supported your study, including funding received from your institution.

: Thank you, this study was granted by the Center of Excellence for the Hill tribe Health Research, Mae Fah Luang University with the No. 1/2021.

b) State what role the funders took in the study. If the funders had no role in your study, please state: "The funders had no role in study design, data collection and analysis, decision to publish, or preparation of the manuscript."

: The funders had no role in study design, data collection and analysis, decision to publish, or preparation of the manuscript. Please kindly see in our cover letter.

c) If any authors received a salary from any of your funders, please state which authors and which funders.

: No. no researchers received a salary from the funder.

d) If you did not receive any funding for this study, please state: “The authors received no specific funding for this work.” Please include your amended statements within your cover letter; we will change the online submission form on your behalf.

: N/A

5. Thank you for stating the following in the Acknowledgments Section of your manuscript: "We thank Mae Fah Luang University and The Center of Excellence for The Hill Tribe Health Research for supporting the grants. " We note that you have provided additional information within the Acknowledgements Section that is not currently declared in your Funding Statement. Please note that funding information should not appear in the Acknowledgments section or other areas of your manuscript. We will only publish funding information present in the Funding Statement section of the online submission form.

Please remove any funding-related text from the manuscript and let us know how you would like to update your Funding Statement.

: Thank you, it’s removed.

Currently, your Funding Statement reads as follows:

"The funders had no role in study design, data collection and analysis, decision to publish, or preparation of the manuscript."

Please include your amended statements within your cover letter; we will change the online submission form on your behalf.

: Thank you, we have mentioned all necessary in the cover letter, please kindly update the information in the system accordingly.

Reviewer #1: This topic would add knowledge to stigma-related studies. Nevertheless, the manuscript needs tremendous flow, coherency, and sentence structure improvement. The current writing style needs to be more scientific. A professional English editing service is deemed necessary.

1.The introduction did not clearly justify the rationale on the conduct of this study.

: We have seriously and heavily revised the whole section of the introduction. Thank you very much in all the comments along every sentence and paragraph.

2.The method need to be explained explicitly.

: Thank you, we have extended details as necessary, thank you so much.

3.The discussion is not adequate.

: Thank you, we have added as needed in this part, thank you.

4.The recommendation in the conclusion appeared abruptly.

: Thank you for the comment, we have seriously discussed in the recommendation, however with the context and the system (health system), we would like to maintain our original thought. However, we have revised the section of the conclusion.

Following are the comments and revision on PDF form

5. Introduction: overall, the introduction needed tremendous improvement in terms of presenting and justifying the facts. At the first sentence page 2, please rephrase this sentence. reviewer has difficulty in comprehending this sentence.

: There are rephrased, page 2 lines 24-25.

6. Please rephrase the second paragraph

: It is rephrased, page 2 lines 25-28.

7. Page 3 line 2-3, considering removing this sentence, just end the sentence with citation will do.

: Thank you, it's improved accordingly (page 3, lines 2-4).

8. Page 3 line 3-4, please rephrase

: It's rephrased.

9. Page 3, suggest stating the effect or giving example of these short and long term effect.

: It's improved.

10. Page 3 the second paragraph, please cite references and please rephrase.

: Thank you, it's cited.

11. Page 3, line 9, how many groups? and which group? Not clear to reviewer, please be specific.

: Since our original though, did not go to any specific group of people of the hill tribe. Rather identifying people who were living in specific geographical areas according to the inclusion and exclusion criteria, then we did not mention the specific group.

12. Page 3 line 10, unclear sentence. how often it was?

: It's improved.

13. Page 3 line 11, what are the other characteristics? And how does the hill tribe people be related with the 'other characteristics' and causing them to be stigmatized? the inclusion of hill tribe people is abrupt in this sentence.

: It's rewritten, lines 20-22.

14. Page 3, line 14, indicate or give example of the minority in Thailand.

: It's improved

15. Page 3 line 16, which population? And what factors? please state or elaborate.

: It's improved.

16. page 3 line 21, suggest omitting 'with ... digits'.

: Thank you, it's improved accordingly.

17. page 3 line 22, please rephrase. Sentence structure error.

: Thank you, it's rephrased.

18. page 3 line 24, please quantify or state what kind of healthcare problems?

: It's added information.

19. page 3, line 25, please cite a reference and how does the underlying healthcare problems been associated with stigma? please justify

: It's cited with justifications.

20. page 3 line 26-31, reviewer cannot connect the justification on why the author need to conduct this research. please define adaption at the prior paragraph or sentences. Sudden appearance of 'adaption' could put reader in wonder. what is the linkage of this sentence with the prior and latter sentence?

: Thank you very much, the paragraph and information have been reorganized and added information.

21. page 4 line 1-3, please rephrase this sentence.

: It’s improved

22. page 4 line 5-6, please define phenomenological method and justify the use of this method in your study and this sentence is ambiguous, not clear to reviewer.

: Since the terminology has its definition, we feel that it is not necessary to put any information in this sentence. We are sorry, we would like to maintain its original sentences in this context.

23. page 4 line 8-9, any reason selecting this age range? ,how did the author identified patients had experience of stigma? ,

: We use our inclusion and exclusion to screen those who met the criteria before the interview. To know about having stigma experience, they were explained and helped in identifying their experiences in facing the stigma.

24. And Also, kindly describe the tribes that is target population. A sudden appearance of different tribes at the results part may appear to confusing to some of readers.

: We have more than 15 years of experience in working research on the hill tribe people, then in practice, there were no mistakes. In simple response to the question, identifying the hill tribe we commonly used by asking them. We also do know them by language, dress, housing style, culture, etc.

24. page 4 line 11, please rephrase. And, what are the background of these key-informant?

: It’s rephrased with information.

25. page 4 line 19-20, unclear to reviewer. Please rephrase.

: It’s rephrased

26. page 4 line 25, do state the sampling frame. were these four villages resided by all hill tribes or these four out of XX number were selected due to the geographical location?

: There are 769 hill tribe villages located in Chiang Rai Province, but with the specific qualitative study design, we selected four villages that met the criteria (one located close to health promoting hospital, one located close to a district hospital, and two far away from hospitals and city). People who lived in these four villages and had stigma experienced were purposively selected.

27. page 4 line 30, what language medium was used for the interview session?

: We used Thai, and information was added.

28. page 5 line 3, what was the sampling technique? please describe.

: It’s added, purposively selection.

29. page 5 line 5-6, were each transcript analysed first before proceeding to interview the next participant? please elaborate. And any translation being done? who did the translation? what was the process?

: Since we could complete in a short period for the interviews, then the following processes were operated after the completion of the all interviews.

30. page 5 line 7, by whom? the interviewer?

: By researchers

31. page 5 line 15, do you mean 'those who are illiterate'?

: Who could not write and singe their name, it’s improved

32. page 5 line 18, do you mean audio records?

:Yes, it’s improved.

33. page 5 line 20, all translated quotes need improvement in sentence structure, grammar, etc...

: Thank you, it’s improved.

34. page 5 result, The themes and sub-themes generated were not concise. It appeared raw, and needed a lot of improvement. Please kindly do more literature search to derive more accurate theme and sub-themes. The theme 'responses' had a lot of sub-sub themes. This had confused the reviewers.

: Thank you, we revised the whole process of the analysis (this is the main reason for taking a long time to revise the manuscript). We found that we have made the proper and accurate findings. However, in the term “Response”, we changed to “Reaction to the experience” which is what we want to say.

35. page 5 line 24, please check if this is an appropriate way to report the min and max. and what is the unit, was this in percentage?

: Thank you, its’ revised and put in the proper place.

36. page 5 table 1, sex, suggestion: gender instead of sex

: Thank you, it’s changed.

37.page 5 table 1 age, any reason to categorise the age category as such?

: We used our experience base while conducting a quantitative study.

38. page 5 table 1, age , suggest presenting these number in the main text instead of adding into the table. please delete this row from the table

: Thank you, it’s improved

39.page 5 table 1, monthly income, present in the main text instead of table

: Thank you, it’s improved

40. page 7 line 10, kindly define form of stigma.

: Since it is in the result section, and it is widely used, including the surrounding context is saying what is the definition. Then we would like to maintain the original form. We do hope you understand.

41. page 7 line 16-17, please define verbal form of stigma. and cite reference for 'major form of stigma' and not clear to reviewer

: Since it is in the result section, and it is widely used, including the surrounding context is saying what is the definition. Then we would like to maintain the original form. We can’t put any reference into this section (result) as scientific writing. We do hope you understand.

41. page 7 line 25, was expression a form of verbal language?

: Yes, it is.

42. page 7 line 27-28, rephrase and the translated verbatim needed improvement.

: It’s rephrased and improved.

43. page 8 line 1, please check the sentence structure.

: : It’s improved.

44. page 8 line 22, define physical form of stigma

: Since it is in the result section, and it is widely used, including the surrounding context is saying what is the definition. Then we would like to maintain the original form. We can’t put any reference into this section (result) as scientific writing. We do hope you understand.

45. page 9 line 28, please define. was negative look a proper theme? please conduct more literature search

: Thank you for the comment, after we carefully understand the context, we would like to use the original term. Since in different contexts of experiences, using the proper terms to reflect the situation would be the best choice. We do very much hope you understand us.

46. page 11 line 24, please rephrase. having difficulty in understanding this sentence.

: It's rephrased

47. page 13 line 15, please define. Reviewer have difficulty in understanding the themes and subthemes of 'responses'

: We changed to "Reaction to the experience"

48. page 14 line 21, the sub-theme is same as the main theme, i wonder was the analysis was conducted accurately?

: Thank you for the comment, we have revised the whole process of the analysis, and forming the findings. We would like to confirm that we have got the proper forms of the findings. We do hope you understand us.

49. page 18 line 5, please rephrase. additionally, the description and quote of the participant did not reflect this sub-theme.

: It's rephrased, and double checked the contexts including the "quotes"

50. page 18 line 24-27, this paragraph should be in the discussion.

: We have spent a long time to consider your suggestion, however, in this statement we wanted to make a small conclusion about the theme presented which we put the small conclusion in every them presented. Then we would like to maintain its original form. We very much hope that you understand our purpose in putting this sentence here.

51. page 26 discussion, overall, the discussion repeated the study findings. There is a need to improvement of the discussion.

: We have revised the whole section and maintained only the necessary and proper sentences. Thank you for the great comment.

The comparisons of other study findings were not relate to this study findings.

: All the discussion sections have been reviewed, improved, and reorganized in some paragraphs. Thank you so much for the great comments.

The recommendations to reduce stigma were not added in the discussion. The relevant recommendation added abruptly in the conclusion.

: We totally agree with you and have deleted the sentences. Some information has been placed in the ethical section and data collecting section properly.

52. page 19 line 9, experienced instead of detected?

: Thank you, its replaced.

53. page 19 line 12, language barrier?

: Thank you, it’s improved.

54. page 19 line13-19, this is repetition of the results. Suggest author to summarise the findings in a couple of sentence instead of repeating.

: Thank you for the excellent comment, we have revised the whole section.

55. page 19 line 20, how does these two perceptions related to the negative looks express? lack of coherency.

: We have carefully considered, reorganized, and rewritten these sentences.

56. page 19 line 22-25, how does this finding relate to your study findings?

: Thank you, we have put some missing information into the context and re-read its original text to make sure that we have cited and mentioned properly.

57. page 19 line 27-31, How does this relate to your study findings and your prior sentence? And this two sentences should be put under introduction. This is a justification on why you need to conduct this study.

: Thank you so much, we have revised these whole sentences.

58. page 20 line 3-4, please rephrase.

: It’s rephrased.

59. page 20 line 5, a repetition of result.

: Thank you, we agree with you and deleted.

60. page 20 line 8, so, how does this relate to your study findings?

: Thank you so much, we have revised the context and this whole sentence.

61. page 20 line 12, how does this relate to the study finding. The subject of this paragraph is about language barriers. Isn't it?

: Thank you so much for the great comment. We have revised these senses.

62. page 20 line 18-19, please rephrase

: It’s rephrased.

63. page 20 line 28 , any reference to support or justify the discussion of this paragraph?

: Since we said that no reference mentions about that then we cannot cite any reference, we would like to maintain our original form.

64. page 21 line 3-6, please cite a reference for this discussion. if there is no evidence to support this, please state, 'based on the researcher's experience or observation"

: Thank you for the great idea to put this sentence, we have put on it.

65. page 21 line 8, over concluding.

: We have seriously consideration on your comment, however, we found that our statement is not over conclusion. We are sorry and would like to maintain our original sentences.

66. page 21 line 9-16, this is not study limitation. This is informed consent taking process and should be addressed under ethical consideration.

: Thank you we totally agreed with you. The sentences are deleted and essential information has been put into the ethics section.

67. page 21 line 18-22, this is data collection strategy and it should be put under method instead.

: Thank you we totally agreed with you. The sentences are deleted and essential information has been put into the data collection section.

68. page 21 line 25-27, reviewer having difficulty in understanding this sentence. please rephrase.

: It’s rephrased.

69. page 21 line 30-31 and page 22 line 1-4, These recommendation appear abruptly in conclusion. Please discuss the recommendations to reduce stigma in the discussion, whichever is deemed necessary.

: We have discussed this among our research team and concluded that our recommendations are good and reflect Thailand’s health system and context. We do hope you understand us.

Please be kindly informed that since a large part of the manuscript has been revised and improved, then some comments might be deleted for a better statement.

Reviewer #2: Very great effort in bringing this paper, and paper looks wrote with the fashion and interest.
Some comments,

1. Stigma and discrimination is two different concepts with thin line difference like stigma is more with thought process and discrimination is behaviour or action. Most of your results section falls in discrimination aspects than limiting to stigma hence relook into the concepts once again.

: Thank you for the great comment. We have reviewed and understood all relevant information including stigma and discrimination at the beginning and during the study implementation to make sure that we were focusing on stigma. Then all interviews were based on thought and perceived experiences. However, many of stigma experiences are always extending to discrimination. However, we can confirm that we looked only at stigma because before implementing the project we investigated and found that very few experiences of discrimination were detected in these populations. Then we decided to work on stigma.

2. Difference of information on participant selection: earlier authors mentioned person who experienced stigma has been interviewed (How did you screened them before selection please describe), then you mention inclusions, people who attended health care at least once or twice in last year was recruited so little this section is little confusing so think on it.

: Thank you, all paragraphs in the method section have been revised and re-organized.

3. You mentioned about the participants selection process however some methodological perspectives missing in terms of sample, sampling techniques etc.

: Thank you, we have added information on sample and sampling techniques, please see the first paragraph of “methods”.

4. You have collected great response participants however interview guide looks very simple and having many closed ended question so justify how you were able to extract more response from participants.

: Thank you for the great notice. During the question guide development, we had a few ideas to form the questions. However, with the experiences of interviewers (researchers) including the key informant selected to provide information in stressful and painful points, then we got a lot of information during the interview, particularly by probe questions.

5. Please describe the inclusion criteria.

: Thank you for the comment, please see page 4.

6. Author mentioned reinterview was carried out- There would be possibility of bias hence justify this section.

: After reviewing the whole process again, we found that only two people confirmed their information after the completion of the interview. Then we would like to delete this sentence to reduce the confusion for readers.

7. Data saturation: provide details on How many researcher and their expertise.

: Thank you very much, we have added information on the number of researchers and their experiences.

8. Small error in table one entry please cross check- Misplaced with row.

: Thank you so much, it is the numbers of Akha and Lahu and improved.

9. In result a. verbal theme first quote looks more of language barrier than stigma so relook into it.

: Thank you with great notice. We have discussed the point and agreed with you. It's deleted.

10.In conclusion, author had mentioned about three forms of stigma which may confuse the reader since we have other types of stigma and discrimination like self, family, public, structural hence i recommend to rewrite this line or statement.

: Thank you very much, it's rewritten in the whole section of the conclusion.

Thank you so much.
TK
Assoc Prof. Dr. Tawatchai Apidechkul, MSc (Infectious Epidemiology), Dr. P. H (Epidemiology)
School of Health Science, MFU
Director, Center of Excellence of Hill Tribe Health Research, WHO-CC
Former Hubert H Humphrey Fellow (2013-2014), Emory University
Global Health Delivery Intensive (Harvard School of Public Health)
