# Supplementary material for: Stigma experiences and adaptations in accessing healthcare services among hill tribes in Thailand: A qualitative study
Source: PLoS One. 2025 May 2;20(5):e0321119. doi: 10.1371/journal.pone.0321119 (PMC12047764; doi:10.1371/journal.pone.0321119)
Supplement: Attachment — Submitted filename: Point-by-point respone to the comments.pdf [file pone.0321119.s006.pdf]

Respond to editor and reviewer's comment

Thank you so much for the opportunity; we have improved all points of your concern. The manuscript has also improved its English by AJE, which is the second time. We do very much hope that you are happy with this version.

We invite you to submit a revised version of the manuscript that addresses the points raised during the review process.

1. Kindly revise your Introduction section to provide a proper background on 'stigma" with appropriate references.

Thank you. We have carefully revised the entire introduction section.

2. Please thoroughly revise your Methods section to provide better clarification on 'inclusion and exclusion criteria'; more details on the qualitative analysis process, including how themes and subthemes where derived, methods for resolving intercoder agreements/disagreements, considering that the COREQ checklist mentions there were '21 data coders'?, etc.

: Thank you for the comment, it's revised and improved in the whole section

3. Consider rewriting the Results section based o the themes and themes derived from the study for better flow and clarity.

: This section is analyzed, and revised as suggested wholly.

4. Please submit an updated COREQ Checklist after the above revisions, especially with regards to the qualitative methods used in the study.

: It's submitted together with this revised paper submission.

5. Consider engaging the services of an English language editor to assist with language editing of the manuscript before resubmission.

: The paper has been revised by native speakers.

6. Kindly address all other issues raised by the peer-reviewers.

: We have carefully revised and improved according to all the reviewers' comments.

Reviewer #1: Overall comment: Though the manuscript has improved following round one revision, the writing skill can be improved further. Having professional English editing for academic papers would help tremendously. The current version is too lengthy, probably owing to limitations in writing or language proficiency. The manuscript could be more concise.

: Thank you for the comment. In this version, one more native English speaker helped us in editing the whole paper before submitting it.

Reviewer #3: Reviewer comment on revision number 1:
I find the topic of the study interesting and commend the authors on being able to interview 85 individuals, which is a feat in qualitative studies. With 85 interviews of 45 minutes each, I was expecting much richer discussion and nuanced insights into the stigma experienced by the hill tribe people in Thailand. However, I find that there is a lack of clear understanding of the authors on stigma theory reflected through very little nuances and depth in the introduction, results, and discussion section of the paper. This is somewhat disappointing considering the time and effort of research participants and researchers that have gone into this. Unfortunately, I do not think that this manuscript is fit for publication in its current form in PLOS and would advise authors to consider doing more studies on stigma theories and bringing in more reflections on the results and discussion sections. See below some of my specific comments.

Introduction:
It seems that the authors do not have an in-depth understanding of stigma phenomena, and this is shown in the introduction section. The first sentence provides a definition that does not give a complete picture of stigma as 'negative attitude' is only one aspect of stigma. Their referencing of APA website rather than the most cited definitions in the stigma field provided by Goffman or Thornicroft is reflective of their lack of proper insight into the topic they have conducted the research on. Verbal and physical stigma are generally not the categorization of forms of stigma but manifestations of interpersonal stigma. It is recommended that authors conduct some review of stigma literature and theories to gain more insight into stigma processes.

: Thank you so much for your kind comments. Even I have been trained in a two-week program under the FIC Stigma Research Training program (NIH) in 2021. I also attended several mental health, stigma, and qualitative classes while attending my coursework terms of my Master of Medical Science- Global Health Delivery (MMSC-GHD) at Harvard Medical School (cohort 2023-2025), I agreed with you that I need to learn more to understand the stigma which is very much interested to me.

Methods:
The lack of information in the methods section is concerning.
The authors mention that the inclusion criteria (pg.4, line4) were "hill tribe people aged >=15 years who had attended a hospital at least twice within the previous year and experienced stigma while accessing a service in a hospital. Those who had poor capacity to provide essential information according to the interview guide were excluded from the 7 study" but it is not clear what "poor capacity to provide essential

information” means or who made the decision (and under what criteria) regarding the capacity. Since this is also a qualitative study, the authors fail to reflect on what excluding people from a certain capacity to provide information would mean in terms of inclusivity of research and ethical implications around it.

: Thank you so much for the great comment. We mentioned the exclusion in our proposal, and after careful discussion of the point, we felt that it was not a good step for the qualitative method, and we did not use it when selecting participants. We have deleted this sentence from the paper. We used a purposive method to select the participants with stigma experiences for the interviews.

The authors do not have information on the qualitative analysis process. Information is missing on what analysis method was used, how were the themes derived, how many people were involved in the coding process, how inter-coder agreement was assessed, etc.

: Thank you for the comment. We have extended the content in this section; Please see page 5, lines 1-8.

Results:

In the form of stigma, the authors again talk about verbal, physical, and negative looks. These are examples of interpersonal stigma manifested as negative behavior by health workers towards hill tribe people. This interpersonal form of stigma is reported by the hill-tribe people and so can also be called experienced stigma. It would be better if the authors phrased it as the experienced stigma of hill tribe people while accessing healthcare services. Other forms of stigma that could have been explored by authors and would have been very interesting would have been self-stigma and structural stigma. In addition, the authors mention 4 forms of stigma were identified (pg.7 line 8) but describe only 3.

: The section has been revised and improved.

: It has three forms, and it’s improved.

I find the quotes very interesting, but the entire result section lacks the depth and nuances of themes being discussed that a good qualitative study requires. It seems that the authors have just added quotes one after another without providing any breadth, depth, or nuances of the themes.

: We are so sorry to make you feel like that. We have revised the whole process of the analysis and revised some points that we could do. If you have any suggestions, please do so.

A similar issue is also seen in the discussion section where authors have just regurgitated the results.

: Thank you for the comment. We have revised many points in the discussion section, particularly your comments on the table. Thank you so much, and very appreciated.

1st revision: Stigma experiences and adaptations while accessing health care service among hill tribes in Thailand: a qualitative study

Overall comment: Though the manuscript has improved following round one revision, the writing skill can be improved further. Having professional English editing for academic papers would help tremendously. The current version is too lengthy, probably owing to limitations in writing or language proficiency. The manuscript could be more concise.

Section	Context	Reviewer's Comment
Introduction	Verbal stigma is the form of voice and language used for one's negative attitude to the other [10]. Physical stigma could present in various forms to present one's negative attitude to the other [10].	These two sentences can be merged.<br><br>: Revised, see lines 28-29, page 2
	There is no evidence of stigma among the hill tribes while accessing health services in a health institute.	Please rephrase this sentence<br>:Revised, see lines 20-21, page 3
	-	Overall, please improve introduction, justification on why the researcher need to determine stigma among hill tribes. Professional English editing will definitely help in improving the writing.<br><br>: All sections have been improved.
Result	Negative looks can discourage people, making people think of themselves in negative ways, especially hill tribe people who live in poor communities in Thailand.	This paragraph needed improvement.<br><br>: Revised, see lines 7-9, page 10
	a. Completely not understood message, then no impact received	Could be improved, needed to be more concise<br><br>: Revised, see line 13, page 10
	Little understanding message, then little pain received	Could be improved, needed to be more concise<br>: Revised, see line 10, page 11
	Without effective communication, misunderstandings could impact several things.	What kind of several things? Please describe.<br>: Revised, see line 16, page 11
	c. Fully understood message, then with full impact received	Could be improved, needed to be more concise

		: Revised, see line 32, page 11
	A nonresponse was one of the responses when the patient did not say anything.	This sentence could be improved, try to rephrase it.<br>: Revised see lines 2-3 , page13
	Responses	The subtheme of non-responses could be merged under “responses”.<br>: Thank you for the comment. We, all authors, have discussed in this point seriously, and decided to maintain in current form to reduce the confusing. However, thank you so much for the suggestion.
	-	Overall, the results are clearly described, however, the sub-themes needed to be more concise.<br><br>There are too many sub-subthemes, which could be improved and merged further. The results could be consolidated further.<br>: Thank you for the comment. We took long time looking back to our raw data and repeating the process of the analysis, and at the end we would prefer to maintain the current form of our result presentation. If we merge any part, there will be impact on the raw data and the flow of getting the structure of our findings. We also have tried to give back the final form/ theme to the participants and found that it is perfectly accepted by the participants.
Discussion	Those who did not understand the messages did not get the impact; those who had little understanding of the message got little pain, and those who fully understood the message got full impact.	This sentence is redundant, please improve it.<br><br>: Revised, see lines 12-14, page 18
	The hill tribe members had two main reactions when facing stigma: non-response and response (proper, nonproper immediate response, and aggressive response).	A repetitive of the results.<br>: Revised, see line 14-15, page 18
	Surprisingly, the ability to understand the message could lead to different impacts on the message receivers. If the situation of verbal stigma occurs, those who completely understand the message are fully impacted while those who do not understand the<br>message will have little or no impact.	A repetitive of the results.<br><br>: Revised, see lines 19-20, page 18.
	but the patients did not understand the messages received then these situations could develop intentional and unintentional actions which were one of the sources of stigma.	Please cite a reference for this sentence<br><br>: Revised, see line 28 with the new reference no 29, page 18

	that promoting a good organizational culture and developing healthcare workers' interpersonal skills could minimize the impact of stigma on patients	And hence, how this would imply to the findings of your study. What information or recommendations do you try to convey when basing this reference on your study?<br><br>: Thank you for the comment. This summarize comes from the findings presented that almost the stigma form hill tribe people received are contru0buted by healthcare workers behaviours.
	Different levels of socioeconomic status……Livingston reported that stigma was a primary source<br>of mental health problems	How did these two sentences be relevant to each other?<br><br>: Thank you for great comment. We agree with you that it is a new idea presented which is not clearly relevant to the key message of the paragraph, then we remove it.<br>.
	These characteristics particularly protected the hill tribe people from the stigma encountered when attending a hospital.	Redundant, it is repetitive in the previous sentence.<br>: Revised, see lines 25-27, page 19
	-	Overall, the discussion could be improved, need to have a more in-depth discussion.<br>: Thank you, we have carefully revised in the whole section including all points of your comments
Conclusion	if they do fully understand, they will get full impact.	Sentence hanging. The full impact of what? Please specify.<br>: Revised, line 10, page 20

Thank you so much.

TK

Assoc Prof. Dr. Tawatchai Apidechkul, MSc (Infectious Epidemiology), Dr. P. H (Epidemiology)
School of Health Science, MFU
Director, Center of Excellence of Hill Tribe Health Research
Former Hubert H Humphrey Fellow (2013-2014), Emory University
Global Health Delivery Intensive (Harvard School of Public Health)
Candidate MMSC-GHD (2025), Harvard Medical School, Harvard University
