# Supplementary material for: Stigma experiences and adaptations in accessing healthcare services among hill tribes in Thailand: A qualitative study
Source: PLoS One. 2025 May 2;20(5):e0321119. doi: 10.1371/journal.pone.0321119 (PMC12047764; doi:10.1371/journal.pone.0321119)
Supplement: Attachment — Submitted filename: Respone to reviewers comments.pdf [file pone.0321119.s011.pdf]

Stigma experiences and adaptations while accessing health care service among hill tribes in Thailand: a qualitative study

5th review

Overall comment	There were noticeable improvements in the writing and sentence flow after utilizing English editing services. However, the quality of English writing in scientific papers still leaves room for improvement.
Study title	Query dissolved.
Introduction	A few studies have reported on the stigma experienced and stigma adaptation among the hill tribes while accessing health services in at a health institute.<br><br>**Comment**: Please cite this statement when you mentioned 'a few studies.'<br><br>: References 20 is placed
	A few studies have reported on the stigma experienced and stigma adaptation among the hill tribes while accessing health services in at a health institute.<br><br>**Comment**: please state or describe the findings of these studies specially about stigma adaptation.<br><br>: Accepting the situation and choosing a private clinic were their adaptations (see page 3, lines 23-24)
Sample size	• I understand that the sample size in qualitative research is not pre-determined. However, I would like the authors to clarify the considerations used in estimating the number of participants to be included in the study.<br>In the proposal development and IRB approval stage, we estimated that we needed 60 participants from those who had stigma experiences from accessing healthcare services. This has been added to page 4, line 23.<br>**Comment**: Please read about sample size estimation in qualitative studies. Typically, at least six participants are required for an interview topic, but the ultimate sample size depends on the complexity of the topic. Some references suggest sampling 10 to 15 participants as an estimate for a proposal. Therefore, I assume your 60 samples are based on 15 participants per village. Please use this rationale and cite an appropriate reference to support it.<br>• Additionally, was there any estimation of the number of participants from each village?<br>Yes, we estimated 15 people in each village were selected to be interviewed. This has been added to page 4, line 24.<br>**Comment**: same as above

	:Thank you, we have added information with reference in page 4, lines 26-27.
interviewer	You have 11 interviewers. Did the author check inter-interviewer reliability, or was any training provided to the interviewers for this study?<br><br>Yes, all the interviewers had been trained in conducting indepth interviews and had conducted least three in-depth interviews in project work experience. We all worked together during the proposal concept development, tool development and quality review, and cross-checked the completeness and quality<br><br>during the whole process of data acquisition and analysis.<br><br>**Comment**: please state this sentence in the manuscript 'all the interviewers had been trained in conducting in-depth interviews and had conducted least three in-depth interviews in project work experience.'<br><br>: Thank you, it's added in page 5, lines 5-6.
Saturation point	What approaches were used to determine data saturation? Please specify.<br><br>: We went to the villages several times to complete the interviews with 60 participants. After the visits, we always discussed and recorded the key points. We determined data saturation had been reached when no more critical evidence, thoughts, experiences or possible themes were detected during our discussions.<br><br>**Comment**: please include this statement in the manuscript<br><br>: Thank you it's added in page 5, lines 12-15.
Discussion	
	'These basic profiles of the hill tribe people are working as the original sources of the stigma'<br>Comment: any literature to support this?<br>This is the summary sentence which is essential to complete the expressions in the previous text.<br><br>**Comment**: This is a conclusive statement, but it is not based on your own study findings unless you cite literature to support the claim that isolated geographical location, low health literacy, and/or lower socio-economic status are the primary sources of stigma. Alternatively, you could rephrase your statement to better summarize your study findings.<br><br>: Thank you, it's revised, please see page 19, lines 8-9.

	'To address the stigma issue among the hill tribe people, there should focus on the difference in stigma experienced in accessing different clinics.'<br>Comment: what does this means?<br>It means that, in the interventions or implementations to address the stigma in these populations, the difference forms of the stigma exposed must be considered.<br><br>**Comment**: Please include the rephrased sentence in place of your original one.<br><br>: Thank you, please see page 20, lines 29-30.
