# Supplementary material for: Stigma experiences and adaptations in accessing healthcare services among hill tribes in Thailand: A qualitative study
Source: PLoS One. 2025 May 2;20(5):e0321119. doi: 10.1371/journal.pone.0321119 (PMC12047764; doi:10.1371/journal.pone.0321119)
Supplement: Attachment — Submitted filename: Stigma Thai Hill Tribes.docx [file pone.0321119.s004.docx]

**1^st^ revision: Stigma experiences and adaptations while accessing health care service among hill tribes in** **Thailand: a qualitative study**

Overall comment: Though the manuscript has improved following round one revision, the writing skill can be improved further. Having professional English editing for academic papers would help tremendously. The current version is too lengthy, probably owing to limitations in writing or language proficiency. The manuscript could be more concise.

| Section | Context | Reviewer’s Comment |
| --- | --- | --- |
| Introduction | Verbal stigma is the form of voice and language used for one’s negative attitude to the other [10]. Physical stigma could present in various forms to present one’s negative attitude to the other [10]. | These two sentences can be merged. |
|  | There is no evidence of stigma among the hill tribes while accessing health services in a health institute. | Please rephrase this sentence |
|  | - | Overall, please improve introduction, justification on why the researcher need to determine stigma among hill tribes. Professional English editing will definitely help in improving the writing. |
| Result | Negative looks can discourage people, making people think of themselves in negative ways, especially hill tribe people who live in poor communities in Thailand. | This paragraph needed improvement. |
|  | ***a. Completely not understood message, then no impact received*** | Could be improved, needed to be more concise |
|  | ***Little understanding message, then little pain received*** | Could be improved, needed to be more concise |
|  | Without effective communication, misunderstandings could impact several things. | What kind of several things? Please describe. |
|  | ***c. Fully understood message, then with full impact received*** | Could be improved, needed to be more concise |
|  | A nonresponse was one of the responses when the patient did not say anything. | This sentence could be improved, try to rephrase it. |
|  | Responses | The subtheme of non-responses could be merged under “responses”. |
|  | ***-*** | Overall, the results are clearly described, however, the sub-themes needed to be more concise.  There are too many sub-subthemes, which could be improved and merged further. The results could be consolidated further. |
| Discussion | Those who did not understand the messages did not get the impact; those who had little understanding of the message got little pain, and those who fully understood the message got full impact. | This sentence is redundant, please improve it. |
|  | The hill tribe members had two main reactions when facing stigma: non-response and response (proper, nonproper immediate response, and aggressive response). | A repetitive of the results. |
|  | Surprisingly, the ability to understand the message could lead to different impacts on the message receivers. If the situation of verbal stigma occurs, those who completely understand the message are fully impacted while those who do not understand the  message will have little or no impact. | A repetitive of the results. |
|  | but the patients did not understand the messages received then these situations could develop intentional and unintentional actions which were one of the sources of stigma. | Please cite a reference for this sentence |
|  | that promoting a good organizational culture and developing healthcare workers’ interpersonal skills could minimize the impact of stigma on patients | And hence, how this would imply to the findings of your study. What information or recommendations do you try to convey when basing this reference on your study? |
|  | Different levels of socioeconomic status……Livingston reported that stigma was a primary source  of mental health problems | How did these two sentences be relevant to each other? |
|  | These characteristics particularly protected the hill tribe  8 people from the stigma encountered when attending a hospital. | Redundant, it is repetitive in the previous sentence. |
|  | - | Overall, the discussion could be improved, need to have a more in-depth discussion. |
| Conclusion | if they do fully understand, they will get full impact. | Sentence hanging. The full impact of what? Please specify. |
