# Supplementary material for: Stigma experiences and adaptations in accessing healthcare services among hill tribes in Thailand: A qualitative study
Source: PLoS One. 2025 May 2;20(5):e0321119. doi: 10.1371/journal.pone.0321119 (PMC12047764; doi:10.1371/journal.pone.0321119)
Supplement: Attachment — Submitted filename: Stigma Hill Tribes Thailand .docx [file pone.0321119.s007.docx]

Stigma experiences and adaptations while accessing health care service among hill tribes in Thailand: A qualitative study

4^th^ review

| Overall comment | The study findings serve as valuable input for policy development. However, important aspects related to conducting qualitative research need to be addressed |
| --- | --- |
|  | The quality of English writing in scientific papers remains unsatisfactory. It is recommended to use English editing and proofreading services  : It has been approved by AJE. |
| Study title | Suggestion for improvement in sentence structure: Stigma Experiences and Adaptations in Accessing Healthcare Services Among Hill Tribes in Thailand: A Qualitative Study  : Thank you, it has been revised. |
| Introduction | No scientific evidence has been reported regarding the stigma experienced by hill tribes when accessing health services at healthcare institutions.  **Comment**: A study titled 'Components of Stigma and Its Impact on Maternal and Child Health Services and Outcomes: Perspective of Akha Hill Tribe Women in Thailand' was published in 2022. Please update your literature review accordingly  : Thank you, the sentence has been revised. |
|  | The author may need to include a definition of stigma adaptation in the introduction.  : Information has been added to page 3, lines 23–24. |
| Method | Four villages were selected, but the approach used for sampling these villages needs to be specified. Was purposive sampling employed? What criteria were used to select these four villages?  : This has been clearly defined on page 4, lines 21–23. |
| Interview guide | Improve this sentence: ‘The final question guide consisted of nine questions’  : We have revised the sentence; please see page 4, line 14. |
| Sampling method | What method was used in sampling the participants?  : Purposive sampling was used; please see page 4, lines 23–24. |
| Data collection method | All interviews were recorded after obtaining approval from the participant, and field notes were taken.  Comment: audio was recorded or audio-visual data was recorded? Please specify.  : Audiovisual recordings are not approved for use in any research. We referred to an audio recording, which is the process of capturing sound or speech using a recording device. This is clearly stated on page 5, line 3. |
| Sample size | - I understand that the sample size in qualitative research is not pre-determined. However, I would like the authors to clarify the considerations used in estimating the number of participants to be included in the study.   : In the proposal development and IRB approval stage, we estimated that we needed 60 participants from those who had stigma experiences from accessing healthcare services. This has been added to page 4, line 23.   - Additionally, was there any estimation of the number of participants from each village?   : Yes, we estimated 15 people in each village were selected to be interviewed. This has been added to page 4, line 24.   - Were there any considerations regarding aspects such as age, gender, ethnicity, religions, incomes, or diseases when selecting participants, which might subsequently influence the sample size?   : No. At the beginning, we included the criteria of selecting villages and participants without any additional criteria regarding age, gender, ethnicity, religions, income, or diseases. |
| interviewer | You have 11 interviewers. Did the author check interinterviewer reliability, or was any training provided to the interviewers for this study?  : Yes, all the interviewers had been trained in conducting in-depth interviews and had conducted least three in-depth interviews in project work experience. We all worked together during the proposal concept development, tool development and quality review, and cross-checked the completeness and quality during the whole process of data acquisition and analysis. |
| Saturation point | What approaches were used to determine data saturation? Please specify.  : We went to the villages several times to complete the interviews with 60 participants. After the visits, we always discussed and recorded the key points. We determined data saturation had been reached when no more critical evidence, thoughts, experiences or possible themes were detected during our discussions. |
| Results | "A certain level of improvement in the themes was noted."  The explanations of the sub-themes need improvement for clarity and accuracy, as there are difficulties in understanding the sentence structure. Professional English editing is recommended.  For example: ‘***Proper response:*** In this type of response, the patient responds calmly and directly when a problem arises. Relatives initiated most responses; however, this situation indicates the lower 18 power of the responders to healthcare workers’, which can be improved to a better clarity paragraph:  **"Proper response: In this type of response, the patient addresses problems calmly and directly when they arise. Although most responses were initiated by relatives, this indicates that patients often hold less power in interactions with healthcare workers."**  : Thank you; we all agreed with you and replaced it. |
| Discussion | ‘A large proportion of the hill tribe population has no formal education, which may result in limited access to health care services.’  Comment: How is the lack of formal education related to limited access to healthcare services? The author may want to clarify this connection.  : Thank you. We have improved this sentence; please see page 18, lines 24–25. |
|  | ‘These basic profiles of the hill tribe people are working as the original sources of the stigma’  Comment: any literature to support this?  : This is the summary sentence which is essential to complete the expressions in the previous text. |
|  | ‘To address the stigma issue among the hill tribe people, there should focus on the difference in stigma experienced in accessing different clinics.’  Comment: what does this means?  : It means that, in the interventions or implementations to address the stigma in these populations, the difference forms of the stigma exposed must be considered. |
| Future research or recommendation | Should future research also investigate healthcare providers' perspectives toward the hill tribes?  : Thank you for excellent point; please see page 20, line 16. |
| limitation | Were only literate participants included in the study? If so, this should be highlighted as a limitation, as it may have excluded the perspectives of non-literate individuals who could offer valuable insights.  : No, we did not focus on only literate participants, but rather all people who lived in the selected villages and had stigma experiences. |
