# Supplementary material for: Stigma experiences and adaptations in accessing healthcare services among hill tribes in Thailand: A qualitative study
Source: PLoS One. 2025 May 2;20(5):e0321119. doi: 10.1371/journal.pone.0321119 (PMC12047764; doi:10.1371/journal.pone.0321119)
Supplement: Attachment — Submitted filename: Response to reviewer comment.pdf [file pone.0321119.s008.pdf]

Response to reviewers' comments

Dear Editor and Reviewers,

Thank you so much for the excellent comments and the opportunity to revise this paper. We have carefully revised and improved based on all your comments. Two senior researchers have been asked to provide comments on the findings forming and English. Thank you, and I very much hope that you are happy with this version.

Please use the space provided to explain your answers to the questions above. You may also include additional comments for the author, including concerns about dual publication, research ethics, or publication ethics. (Please upload your review as an attachment if it exceeds 20,000 characters)

Reviewer #1: Overall, I can tell that the authors had put great effort in this research work and manuscript. However, the works particularly the results section remains 'raw' and needed improvement. I understand that the current themes and sub-themes were the agreed upon among the authors, they lack element of conciseness. Considered read up more literatures for improvement of sentence structure, particularly the themes. Analysis of the data was deemed not in-depth. It can be delved further. An experienced senior qualitative researcher might help.

: Thank you for your comment. We have revised the whole data analysis step again to make sure that we do not have any bias during the analyses and pick up only the evidence support without overclaimed interpretation before asking two senior qualitative researchers for their ideas. One of the critical points raised by our senior researchers is the paper is presented in a critical point with a simple presentation of the findings, which will make it easy for those policymakers in Thailand to further action implementation. We have been suggested to maintain the current form of the findings and share them with the policymakers working in the Ministry of Public Health, Thailand and also the WHO staff. They suggested that putting one more interpretation paragraph at the end of the results section would be great. We have put the final interpretation paragraph as suggested (page 18, lines 9-13), I do very much hope that you are happy and agree with us.

English writing skill in scientific paper needed improvement. A native speaker may not be sufficient in english editing of a scientific paper. A professional academic english editor may be necessary.

: Thank you. We have asked two senior researchers to modify the writing in a scientific style and made some modifications. They informed us that language is related to people's cultures, and presenting based on their culture is a beautiful point of a qualitative study.

Reviewer #4: Commendation on revision number 2

Dear Author,

Your manuscript contributes significantly to understanding the discrimination faced by Thailand's hill tribes in healthcare. It identifies the issues and proposes practical solutions. However, I've identified a few areas where improvements can be made to strengthen the paper further:

1. Abstract: In the end, you should restate the main points and highlight the steps that need to be taken. To improve the Hill tribe people, it must emphasize the importance of training healthcare workers, improving communication, and addressing socioeconomic inequalities.

: Thank you for the comment; we have added the information in this section; please see page 2, lines 19-22.

3. Discussion: The demographic information could show why certain groups are important, like why it's interesting that many people don't have high education levels. This will define the context and highlight the significance of these characteristics in discussing stigma.

: Thank you for the critical point of comment. We have added information in page 18, lines 23-27.

3. Limitations and Future Research: The study's weaknesses, including its small sample size or limited geographic focus, and suggestions for future research should be discussed in depth. This would strengthen the academic standard and underscore the need for more research on the topic, thereby motivating further investigation.

: Thank you, we have added information on page 20, lines 15-17.

Thank you,

TK

TK

Assoc Prof. Dr. Tawatchai Apidechkul, MSc (Infectious Epidemiology), Dr. P. H (Epidemiology)
School of Health Science, MFU
Director, Center of Excellence of Hill Tribe Health Research
Former Hubert H Humphrey Fellow (2013-2014), Emory University
Global Health Delivery Intensive (Harvard School of Public Health)

Candidate MMSC-GHD (2025), Harvard Medical School, Harvard University
